# Genetic Characterization and Symbiotic Performance of Soybean Rhizobia Under Cold and Water-Deficient Conditions in Poland

**DOI:** 10.3390/plants14121786

**Published:** 2025-06-11

**Authors:** Riku Watanabe, Maria Daniela Artigas Ramirez, Shin-ichiro Agake, Sonoko Dorothea Bellingrath-Kimura, Sylwia Lewandowska, Yuki Onishi, Yohei Nishikawa, Haruko Takeyama, Michiko Yasuda, Naoko Ohkama-Ohtsu

**Affiliations:** 1 Graduate School of Agriculture, Tokyo University of Agriculture and Technology, 3-5-8, Saiwai-cho, Fuchu 183-8509, Tokyo, Japan; 2 College of Biological Science and Technology, Beijing Forestry University, No. 35, Qinghua East Road, Haidian District, Beijing 100083, China; 3 Center for Applied Biotechnology (CBA), Faculty of Experimental Science and Technology, Department of Biology, University of Carabobo, Valencia 2001, Carabobo, Venezuela; 4 Institute of Global Innovation Research, Tokyo University of Agriculture and Technology, 3-5-8, Saiwai-cho, Fuchu 183-8509, Tokyo, Japan; 5 Leibniz Centre for Agricultural Landscape Research (ZALF), Eberswalder Str. 84, 15374 Müncheberg, Germany; 6 Faculty of Life Sciences, Humboldt-University of Berlin, Unter den Linden 6, 10099 Berlin, Germany; 7 Department of Genetics, Plant Breeding and Seed Production, Wrocław University of Environmental and Life Sciences, Pl. Grunwaldzki 24A, 50-363 Wrocław, Poland; 8 Graduate School of Advanced Science and Engineering, Waseda University, 2-2 Wakamatsu-cho, Shinjuku-ku 162-8480, Tokyo, Japan; 9 Research Organization for Nano & Life Innovation, Waseda University, 513 Waseda Tsurumaki-cho, Shinjuku-ku 162-0041, Tokyo, Japan; 10 Institute for Advanced Research of Biosystem Dynamics, Waseda Research Institute for Science and Engineering, Graduate School of Advanced Science and Engineering, Waseda University, 3-4-1 Okubo, Shinjuku-ku 169-8555, Tokyo, Japan; 11 Institute of Agriculture, Tokyo University of Agriculture and Technology, 3-5-8, Saiwai-cho, Fuchu 183-8509, Tokyo, Japan

**Keywords:** soybean, rhizobium, *Bradyrhizobium*, abiotic stress, cold conditions, water-deficient conditions

## Abstract

Soybeans have been cultivated in Poland for more than 140 years. However, Poland’s cold and water-deficient climatic conditions hinder soybean cultivation. Although the availability of suitable soybean varieties in Poland contributes to meeting the demand for soybean production, it is important to identify rhizobial inoculants in Polish soils suitable for soybean cultivation. In this study, we cultivated soybean varieties (Abelina, Merlin, and Sultana) grown in soils taken from four regions in Poland and isolated 330 strains from soybean root nodules. 16S rRNA gene sequencing identified 49 strains of highly stress-tolerant nodule-associated bacteria, including *Bradyrhizobium*, *Rhizobium*, *Ensifer*, *Tardiphaga*, and *Ralstonia* spp. Several isolates exhibited positive effects on soybean growth under cold and water-deficient conditions. In particular, the isolate *Bradyrhizobium japonicum* PSN49, which is phylogenetically similar to *B*. *japonicum* USDA 123, increased plant biomass and nodule formation in the soybean cultivar Abelina under abiotic stress conditions due to its high nitrogen-fixing activity. Whole-genome comparisons between PSN49 and other *Bradyrhizobium* strains revealed that trehalose biosynthesis genes and cold shock proteins contributed to cold stress tolerance. These findings and the strains identified in this study will enhance soybean production and deepen the understanding of the soybean–rhizobium relationship in Poland.

## 1. Introduction

Soybean (*Glycine max* (L.) Merr.) is one of the most important legume plants worldwide, and its seed contains a high amount of protein. Its origins are in East Asia, including China and Japan. The history of soybean cultivation in Japan dates back to before the common era (BCE); however, in Europe, it was only introduced around the 18th century [1]. In European countries, soybeans are mainly used for livestock feed [2] and green manure rather than human food, chemical [3,4], or pharmaceutical applications [5,6]. The demand for Polish field production is systematically increasing to ensure food security. Moreover, soybeans are receiving considerable attention for plant-based meats because of their health and environmental benefits. Therefore, it is important to increase soybean production [7] and to be independent as a country. According to Lewandowska (2016) [8], for many farmers in Poland, the production of this plant is a “godsend” for defective rotations that mainly consist of cereals, maize, and rapeseed. The inclusion of soybean cultivation in crop sequences is an important and valuable element [9]. In Poland, soybeans have been introduced and tested for more than 140 years; however, the first milestone in cultivating this species was only achieved in the second decade of the 21st century. In 2019, the area of soybean cultivation in Poland was 20,000 ha. This area increased to 80,000 ha by 2024. This rapid growth in recent decades indicates that soybean production and cultivation areas in Poland could further increase in the future.

In Poland, as well as in the European Union (EU), there is a need to provide non-GM feed protein sources. Currently, Poland imports approximately 2 million tons of genetically modified (GM) varieties of soybean meal for a total of EUR 4 billion [7]. In the EU, soybean meal is used as the primary source of plant protein in the feed of livestock, such as poultry and cattle [10]. The EU is the largest importer of soybean meal, accounting for 70% of the high-protein components used in the production of compound feed. This results in a dependence on protein purchased from other countries like the USA, Brazil, and Argentina. Therefore, attempts to cultivate soybeans under Polish conditions are needed. One way to achieve a protein balance is to improve legume yields and, in particular, to improve soybean agronomics in European countries [11,12].

Rhizobia are mainly soil diazotrophic bacteria that form nodules on legume roots and fix atmospheric nitrogen (N_2_). They reduce N_2_ to ammonia and then supply it to their host plants. Nitrogenases, encoded by nif genes, principally *nifHDK*, catalyze this reaction. The rhizobia promote host plant growth, and, in return, the host supplies photosynthesized carbon to the rhizobia. Thus, through legume–rhizobium symbiosis, a process called biological nitrogen fixation is realized, contributing to a reduction in chemical nitrogen fertilization. Most rhizobia belong to *Alphaproteobacteria*, including *Rhizobium*, *Bradyrhizobium*, *Ensifer*, *Mesorhizobium*, and *Azorhizobium* [13,14]. However, some rhizobia are related to *Betaproteobacteria*, such as *Paraburkholderia* and *Cupriavidus* [15,16]. The symbiotic relationship between rhizobia and a host plant strongly depends on host genetics, specificity, and the environment. This relationship is accomplished through molecular signaling. The bacterial *nod* gene cluster is associated with nodule formation in rhizobia [17]. The most common *nod* gene, considered the most important among the more than 20 *nod* genes, is *nodDABC*. Symbiosis is initiated by the binding of flavonoids secreted by the legume roots to the nodD protein.

The interaction between soybeans and rhizobia is well known. *Bradyrhizobium* species (slow-growing bacteria), such as Bradyrhizobium japonicum, *Bradyrhizobium diazoefficiens*, *Bradyrhizobium elkanii*, *Bradyrhizobium yuanmingense*, and *Bradyrhizobium liaoningense*, and *Ensifer* species (fast-growing bacteria), such as *Ensifer fredii*, are mainly available as commercial soybean inoculants [18,19,20,21,22]. Currently, imported inoculants are widely used in Poland. However, soybeans with these inoculants form few root nodules, leading to a decrease in the soybean–rhizobia interaction efficiency and, consequently, lower yields under severe cold and water-deficient conditions. Numerous studies on soybean–rhizobium symbiosis and cold or drought stress have been conducted, mainly in countries with severe climates. In Canada, a country with a severely cold climate, a locally adapted rhizobium, *B. japonicum* 532C, is available as a cold-tolerant commercial soybean rhizobium inoculant [23]. *Bradyrhizobium* sp., isolated under arid conditions in Argentina, was found to improve soybean production compared to the commercial inoculant *B. japonicum* E109 [24]. In Germany, located to the west of Poland, soybean rhizobia (*Bradyrhizobium* and *Rhizobium* spp.) exhibit a high biomass and nitrogen fixation activities [25,26]. Therefore, it is important to introduce and develop rhizobial inoculants that are suitable for the local environmental conditions and cultivated varieties. According to an American study, the success of soybean growing depends on a combination of weather conditions, soil type, and cultivar [27]. The identification of a suitable cultivar that tolerates unfavorable weather conditions is essential to promote further progress in breeding work [28]. By developing new varieties adapted to the Polish soil and climatic conditions, soybean meal imports can be reduced. Recently, the growth and yield of soybeans in the cold climates of Poland have been verified [29]. Abelina produces relatively high yields in cold environments. Abelina and Merlin, originally Austrian (AT) cultivars but reproduced in Poland, are currently the most popular soybean varieties in Poland. The cultivar Merlin was the most popular variety grown in Poland during 2010–2022. However, no rhizobial inoculants have been developed for these soybean cultivars in Poland.

This study aimed to determine the best native strains for use as novel soybean inoculants in Poland. Therefore, we isolated and identified locally adapted rhizobia suitable for the Polish environment and screened for strains with the highest symbiotic performance, abiotic stress tolerance, and nitrogen-fixing activity. Whole genome sequencing was also performed on the most promising strain to gain insight into the basis of its growth promotion in severe environments.

## 2. Results

### 2.1. Isolation and Phenotypical Characterization of Rhizobia from Polish Soils

**Figure 1:**
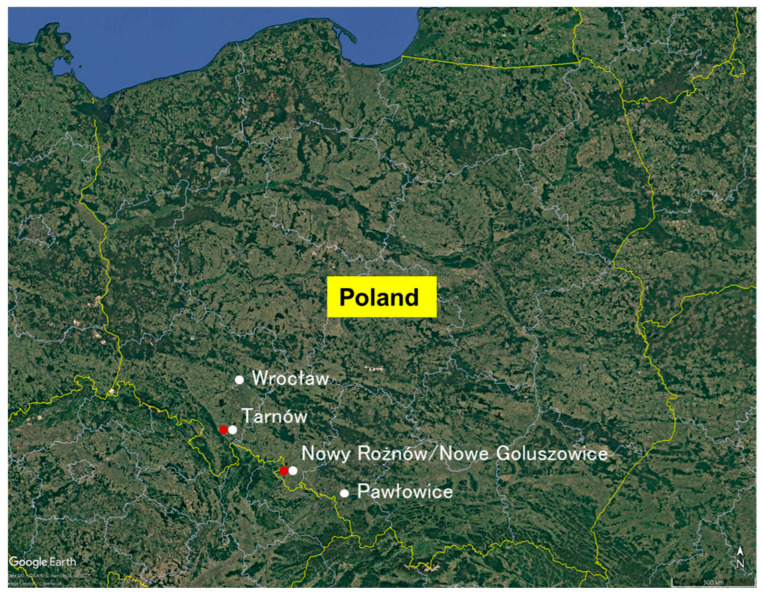
Soil sampling sites. The red dots indicate sites with a soybean cultivation history; the white dots indicate sites with no soybean history. This map was created using Google-Pro Earth, 2023.

The soybean cultivars Abelina, Sultana, and Merlin were grown to isolate abiotic stress-tolerant rhizobia from soils in Poland (Figure 1, Table 1). In total, 330 strains of indigenous nodule-associated bacteria were isolated from the soybean root nodules. Most of the isolates were obtained from fields with a history of soybean cultivation. Only a few rhizobia were isolated from fields without a history of soybean production. Rhizobia could not be isolated from soil sample nos. 3, 4, 5, and 7 (Table 1 and Table S1). After cultivation, plants in the field with no history of soybean growth had yellow leaves, similar to the non-inoculated plants; however, plants in the field soil with a history of soybean cultivation were greener (Figure S1).

### 2.2. Environmental Stress Potential of Polish Rhizobia

**Figure 2:**
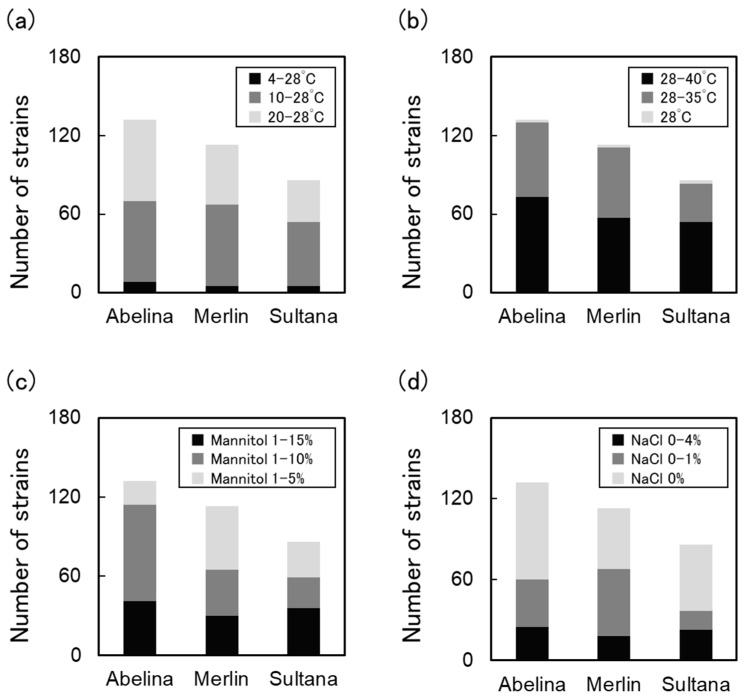
Abiotic stress tolerances of native isolates from Poland. Evaluation of stress tolerance of strains for each isolated host plant. Number of strains for each condition that survived in each medium is shown. (**a**) Cold stress, (**b**) high-temperature stress, (**c**) osmotic stress, and (**d**) salinity stress.

To screen for rhizobia that can endure severe abiotic stress in Poland, the tolerance of the 330 strains was evaluated (Figure 2). Some strains showed an extremely high tolerance to cold, osmotic, and salinity stresses: 18 strains were cold stress-tolerant, 107 were osmotic stress-tolerant, and 66 strains were salinity stress-tolerant. Approximately 86% of the strains isolated from Abelina root nodules survived on YMA containing 10% mannitol, whereas only 58% and 69% of Merlin- and Sultana-isolated rhizobia, respectively, displayed tolerance.

### 2.3. Phylogenetic Characterization of Rhizobia

**Figure 3:**
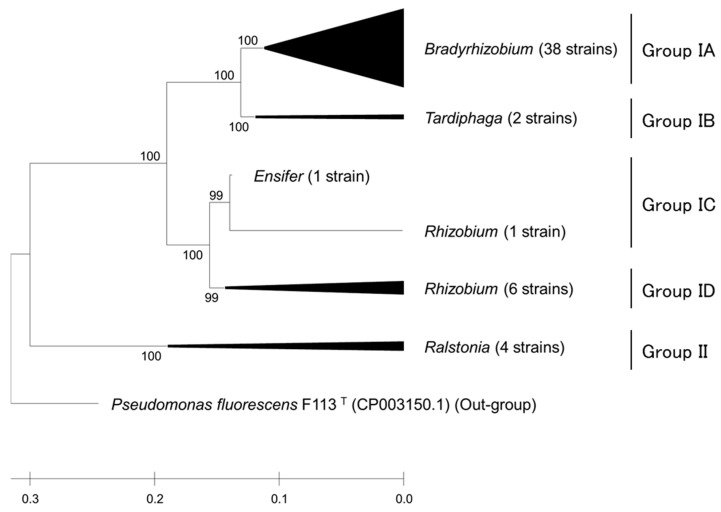
Phylogenetic clustering tree based on 16S rRNA gene sequences of forty-nine representative strains. Labels show cluster genus distribution of selected isolates. Group I includes *Alpha-proteobacteria*, and Group II includes *Beta-proteobacteria*. The length of each sequence is 1366 bp. Tree was constructed using maximum likelihood method. Bootstrap values are shown as percentages from 1000 replicates.

Forty-nine strains were selected as the top-performing strains based on their origin and stress tolerance. Based on 16S rRNA gene sequencing, the representative strains were separated into two groups (I and II; Figure 3). Group I, *Alphaproteobacteria*, comprised four subgroups: Group IA, containing *Bradyrhizobium*; Group IB, containing *Tardiphaga*; Group IC, containing *Ensifer* and *Rhizobium*; and Group ID, containing *Rhizobium*. Group II, containing *Ralstonia*, was classified as *Betaproteobacteria*. *Bradyrhizobium* was identified as the most abundant genus (71%, 35 strains). Both strains of *Tardiphaga* were isolated from soil samples without a history of soybean cultivation (soil sample nos. 1 and 6; Table S2). Most *Bradyrhizobium* (34 strains) samples were isolated from fields with a history of soybean cultivation. *Tardiphaga* and *Ralstonia*, which are not known as rhizobia, were excluded from further assays. Based on the stress tolerance potential and 16S rRNA gene sequences, nine strains were identified as promising strains for further MLST and plant assays.

### 2.4. Nitrogen Fixation-Associated Gene Sequencing and MSLT in Rhizobia

Forty-four representative strains were further evaluated for nitrogen fixation-related genes (*nodD*, a nodulation protein D gene; *nifH*, a nitrogenase reductase gene) and subjected to phylogenetic analysis. The *nodD* and *nifH* genes were amplified in 39 and 43 strains, respectively. The *nodD* of the strains was divided into Group I (containing *B. japonicum* USDA 6^T^) and Group II (containing *Rhizobium etli* CFN 42^T^). The *nodD* gene of most strains (34 strains, 87% of the amplified genes) was classified as Group I and was orthologous to *B*. *japonicum* USDA 6^T^ (Figure S2). *Rhizobium* PMN31 also showed the presence of the *nodD* gene, which was orthologous to *B*. *japonicum* USDA 6^T^. The *nodD* gene was not amplified in PAP2, PAT8, PAN13, PAN21, and PMN27.

Similar results were observed for *nifH* as for *nodD*. The *nifH* genes were sorted into three groups: Group IA, which contained *B*. *japonicum* USDA 6^T^; Group IB, which contained *B. amphicarpae* 39S1MB^T^; and Group II, which contained *R. etli* CFN 42^T^ (Figure S3). Almost 84% of the amplified genes (36 strains), including those of *Rhizobium* PAN13 and PMN31 and *Ensifer* PAN21, were genetically similar to *B*. *japonicum* USDA 6^T^. The *nifH* gene of PMN27 was not amplified.

Housekeeping genes of the nine strains selected as promising bacteria were analyzed (Figure S4). Classification based on the concatenated sequences showed that all five *Bradyrhizobium* strains were phylogenetically similar to *B. japonicum* USDA 123.

### 2.5. Plant Inoculation Assays

To evaluate symbiotic performance, nine representative strains—five *Bradyrhizobium*, three *Rhizobium*, and one *Ensifer*—were selected based on the 16S rRNA gene sequence and inoculated onto the host plant, soybean. Three European soybean cultivars—Abelina, Merlin, and Sultana—were used for the assay. Upon inoculation of the isolated rhizobia in Abelina, one of the most popular cultivars in Poland, all strains induced soybean nodule formation and nitrogen fixation (Table 2). Compared to the positive control, namely, *B. diazoefficiens* USDA 110^T^, inoculation with strains other than PAN21 and PMN31 resulted in comparable increases in plant biomass. Of note, *B. japonicum* PSN49 showed the highest plant growth-promoting effects, with an increase of 67.7% in root dry weight and 17% in whole plant biomass, compared with plants inoculated with *B. diazoefficiens* USDA 110^T^. *B. japonicum* PMN37 and PMN35 induced a significant increase in the nodule dry weight in Abelina. The number of nodules in Abelina increased upon inoculation with *Ensifer* sp. PAN21.

Similarly, for other soybean varieties, inoculation with several isolates, such as PSN49, resulted in increases in biomass similar to that induced by *B. diazoefficiens* USDA 110^T^ (Tables S3 and S4). However, no significant increase was observed in Merlin, except for the number of nodules when inoculated with PAN13. In Sultana, inoculation with PMN31 and PMN30 significantly increased nodule dry weight, while inoculation with PMN30 also led to a significant increase in the number of nodules and acetylene reduction activity.

### 2.6. Plant Inoculation Assays Under Cold Conditions

**Figure 4:**
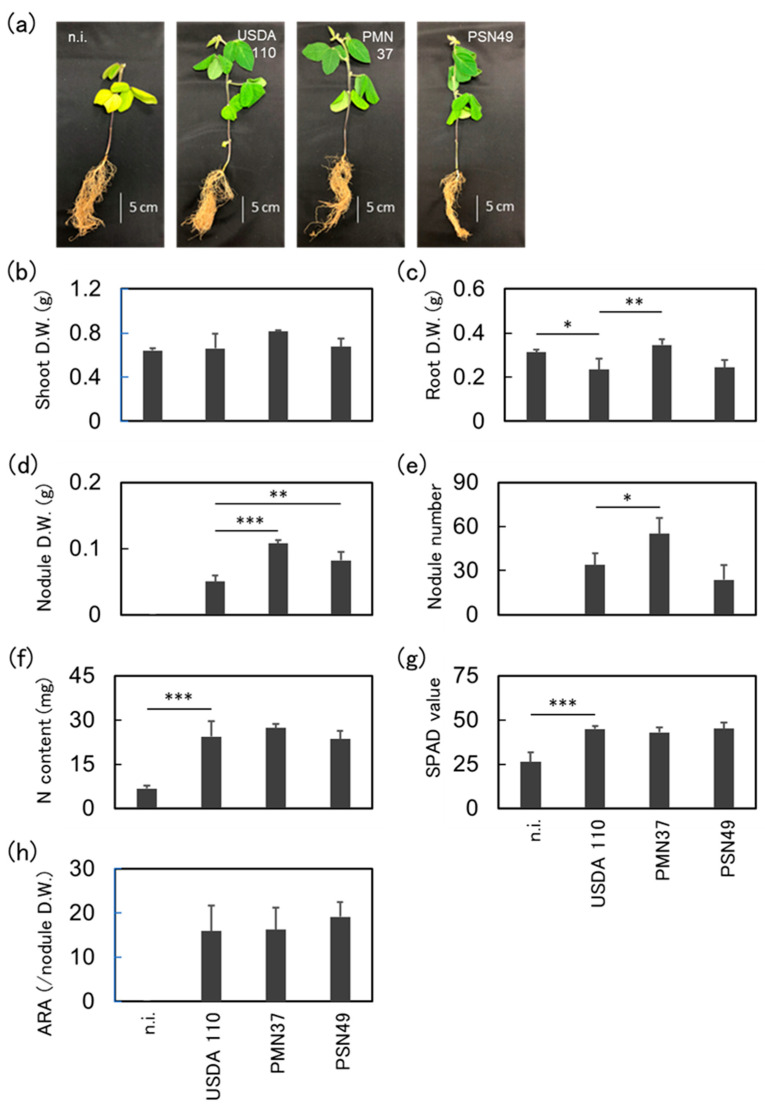
The performance of isolates native to Poland using the Abelina cultivar under cold conditions. Four strains were used as the inoculant for Abelina. (**a**) Plants 6 weeks after inoculation with n.i., USDA 110, PMN37, and PSN49. (**b**) Shoot dry weight, (**c**) root dry weight, (**d**) nodule dry weight, (**e**) nodule number, (**f**) nitrogen content in the shoots, (**g**) SPAD value, and (**h**) acetylene reduction activity (µmol h^−1^ g^−1^ nodule dry weight). “n.i.” indicates non-inoculated plants (negative control). “USDA 110” indicates *Bradyrhizobium diazoefficiens* USDA 110 (positive control) treatment. The values and error bars represent the mean and standard deviation of four biological replicates. The experiment was repeated independently with consistent results. The results for each strain were compared with those for strain USDA 110 using Dunnett’s test; * *p* < 0.05, ** *p* < 0.01, *** *p* < 0.001.

Based on the plant inoculation tests under normal environmental conditions, two strains of *Bradyrhizobium* (PMN37 and PSN49) were selected for pot tests under low-temperature conditions. As shown in Figure 4a, plants with adequate nitrogen fixation maintained their green color, whereas the non-inoculated plants turned yellowish. *Bradyrhizobium* strains had a stable and positive effect on Abelina. Plants inoculated with *B. japonicum* PSN37 had an increased shoot biomass compared to *B. diazoefficiens* USDA 110^T^, but the difference was not significant (Figure 4b). Moreover, inoculation with *B. japonicum* PSN37 led to a significant increase in the root and nodule dry weights of Abelina (Figure 4c,d, and Table S5). Inoculation with *B. japonicum* PSN49 also caused a significant increase in nodule dry weight. However, the two *Bradyrhizobium* strains did not alter the nitrogen content, SPAD value, or acetylene reduction activity of the plants (Figure 4f–h).

PMN37 and PSN49 promoted Merlin and Abelina growth. Plants inoculated with PMN37 or PSN49 showed increased shoot and root dry weights and nodule numbers; however, the differences were not significant (Figure S5a,b,d). Plants inoculated with PSN49 showed significantly higher nodule dry weights than those of *B. diazoefficiens* USDA 110^T^ (Figure S5c). PMN37 also resulted in a high nodule dry weight for Merlin. However, the isolates did not contribute to an increase in the SPAD and acetylene reduction activities (Figure S5e,f).

### 2.7. Plant Inoculation Assays Under Water-Deficient Conditions

**Figure 5:**
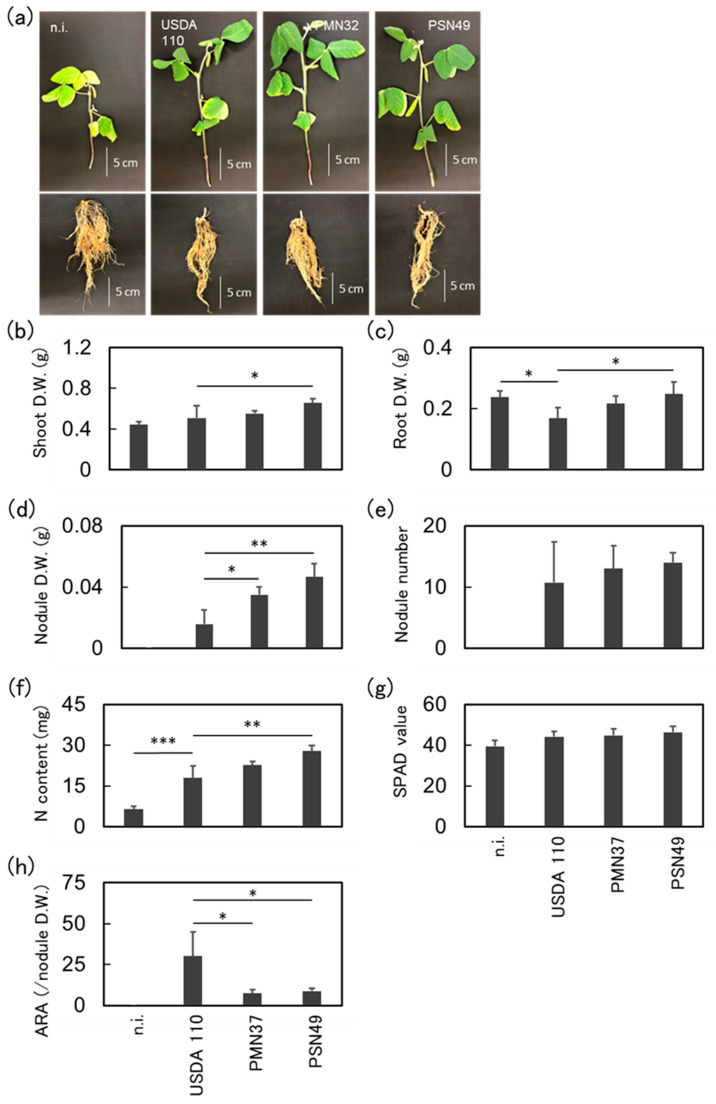
Plant response to inoculation with Polish isolates under water-deficient conditions. Four strains were used as the inoculant for Abelina. (**a**) Plants 6 weeks after inoculation with n.i., USDA 110, PMN37, and PSN49. (**b**) Shoot dry weight, (**c**) root dry weight, (**d**) nodule dry weight, (**e**) nodule number, (**f**) nitrogen content in shoot, (**g**) SPAD value, and (**h**) acetylene reduction activity (µmol h^−1^ g^−1^ nodule dry weight). “n.i.” indicates non-inoculated plants (negative control). “USDA 110” indicates *Bradyrhizobium diazoefficiens* USDA 110 (positive control) treatment. The values and error bars represent the mean and standard deviation of four biological replicates. The experiment was repeated independently with consistent results. The results for each strain were compared with those for strain USDA 110 using Dunnett’s test: * *p* < 0.05, ** *p* < 0.01, *** *p* < 0.001.

Plant assays were performed under water-deficient conditions with the same four strains used in the cold conditions. After 6 weeks of cultivation, leaf color was not as markedly different from that noted under cold conditions (Figure 5a). Under water-deficient conditions, plant growth was greatly enhanced by *B. japonicum* PSN49 inoculation, unlike what was noted under the cold conditions. PSN49 had a significantly positive effect on the shoot dry weight, root dry weight, nodule dry weight, and nitrogen content of Abelina compared to *B. diazoefficiens* USDA 110^T^ (Figure 5b–d,f; Table S5). The nodule dry weights of plants inoculated with PMN37 and PSN49 were significantly increased (Figure 5d).

The root dry weights of Merlin increased significantly upon non-inoculation (n.i.) and inoculation with PMN37 (Figure S6b). Inoculation with PSN49 or PMN37 tended to induce an increase in nodule dry weight compared with *B. diazoefficiens* USDA 110^T^; however, no differences in shoot biomass or SPAD values were observed (Figure S6c). Similar to Abelina, PSN49 and PSN37 exhibited strong growth-promoting effects in Merlin.

### 2.8. Whole Genome Sequencing of PSN49

Whole genome sequencing was performed on the PSN49 strain, which promoted soybean growth under the abiotic stress conditions. The PSN49 genome is composed of 11,082,922 bp of DNA, containing 10,931 CDSs (Table 3 and Table S6). In addition to MLST, orthoANI value calculations (https://www.ezbiocloud.net/tools/ani, accessed on 15 December 2024) of other *Bradyrhizobium* strains and phylogenetic analysis showed that PSN49 is a strain that is genetically close to *B. japonicum* USDA 123.

Cold or drought stress-associated genes are listed in Table S7 given that PSN49 contributed to soybean growth promotion under abiotic stress conditions. *AcdS* (1-aminocyclopropane-1-carboxylate (ACC) deaminase) was annotated as a gene associated with plant growth promotion under abiotic stress conditions. The cold stress adaptation-associated genes were also annotated as follows: *cspA* (cold shock protein), *gyrA* (DNA gyrase), *clpBP* (protein folding-associated genes), *dnaK* (heat shock protein 70), *rnr* (ribonuclease R), and DEAD-box protein-coding genes *deaD*, *rhlB*, *dbpA*, and *rhlE*. The PSN49 genome contained sixteen copies of *cspA* genes, while the *B. diazoefficiens* USDA 110^T^ genome only possessed twelve copies. Moreover, *dbpA* was annotated in the PSN49 genome, coding for a DEAD-box family ATP-dependent RNA helicase. A comparison of the OrthoFinder results between the PSN49 and *B. diazoefficiens* USDA 110^T^ genome and the NCBI database showed that *B. diazoefficiens* USDA 110^T^ has no dbpA-coding gene. PSN49 genome annotation indicated the presence of the following osmotic stress adaptation-associated genes: cell membrane adaptation-associated genes (*desR* and *fabF*), an ectoine synthase gene (*ectC*), proline synthase genes (*proABC*), trehalose synthase-associated genes (*otsAB* and *treSYZ*), trehalose synthase genes (*betAB*), and polyamine synthase-associated genes (*hss*, *arcA*, and *speCEF*). According to OrthoFinder and the NCBI database, the desR-coding gene (*desR*), which encodes a transcriptional regulatory protein, is present in the PSN49 genome but not in the *B. diazoefficiens* USDA 110^T^ genome. The following oxidative stress adaptation-associated genes were also annotated: superoxide dismutase (*sodB* and *chrC*), catalase (*katAEG*), and glutathione S-transferase (*gstB*).

**Figure 6:**
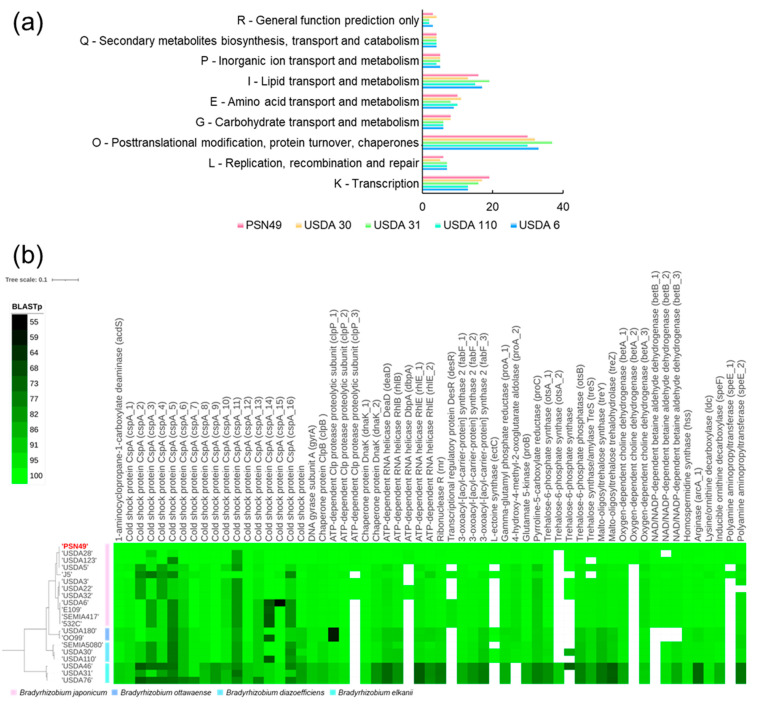
Genome sequencing of *Bradyrhizobium japonicum* PSN49. (**a**) COG of the annotated genes associated with cold and drought stress in PSN49 and *Bradyrhizobium* strains. USDA 30, *Bradyrhizobium diazoefficiens* USDA 30; USDA 31, *Bradyrhizobium elkanii* USDA 31; USDA 110, *B. diazoefficiens* USDA 110; USDA 6, *B. japonicum* USDA 6. (**b**) The identity rates (%) of the annotated proteins associated with cold and drought stress in PSN49 are compared to those in *Bradyrhizobium* strains using UBCG v2 and BLASTp. Dark green to light green, 55–100% identity; blank, <55% identity.

These genes were classified into COG categories with the orthologues of *B. diazoefficiens* USDA 30 and USDA 110^T^, *B. elkanii* USDA 31, and *B. japonicum* USDA 6^T^ (Figure 6a). Compared to the other strains, PSN49 and *B. diazoefficiens* USDA 30 had more genes associated with carbohydrate transport and metabolism (G), including genes involved in the trehalose synthesis pathway. In the transcription category (K), PSN49, *B. diazoefficiens* USDA 30, and *B. elkanii* USDA 31 had a higher number of genes than the other strains. Genes encoding cold shock proteins accounted for most (84.6–94.1%) of this category. A total of 59 representative proteins in the genome of PSN49 were selected and reported in Table S7; the similarity of the proteins to those of nineteen *Bradyrhizobium* strains was evaluated using BLASTp (Figure 6b). The trehalose-6-phosphate synthase protein in PSN49 was notably different from the other strains. This genome contained three copies of this gene, including one orthologous to that of all strains (80.4–100% identity); one orthologous to that of *B*. *elkanii* USDA 31, USDA 46, and USDA 76^T^ (91.32% identity); and another orthologous to that of several strains of *B*. *japonicum* (100.00% identity), *B*. *diazoefficiens* USDA 30 (85.56% identity), and *B. elkanii* USDA 46 (71.72% identity). Proteins with a high identity to dbpA and desR were not identified in some *B. japonicum* and other *Bradyrhizobium*. Only a single copy was present in the genome of *B*. *diazoefficiens* USDA 110^T^. In addition, *B*. *diazoefficiens* USDA 110^T^ lacked orthologues to *clpP*_3, *dbpA*, *desR*, *betA*_2, *betB*_1, and *speE*_1 of PSN49, although several other strains possessed them.

## 3. Discussion

### 3.1. Diversity of the Soybean Rhizobia Isolated from Polish Soil with Inoculant History

In the present study, we isolated rhizobia from 19 soil samples in western Poland using three varieties of soybeans (Abelina, Sultana, and Merlin) as host plants. A clear difference was observed in the number of nodules formed between fields without a history of soybean cultivation (soil sample nos. 1–9) and those with a history (soil sample nos. 10–19). Consistent with Yuan et al. (2020) [26], the number of nodules formed in the soil from fields with a history was large compared to those without a history (Figure S1; Table S1). In fields with a history, a *B. japonicum* inoculant was used as the symbiotic partner. In Poland, *Bradyrhizobium* rhizobia have been used as a viable inoculant for more than 7 years, or even 20 years in some locations, in soybean fields cultivated without the use of additional inoculants [33,34]. Past soybean cultivation and the application of rhizobial inoculants potentially affected the community structure of soil rhizobia and increased the survivability and priority of soybean rhizobia in the soil, even under cold and water-deficient conditions in Poland. Furthermore, these strains were considered environmentally adapted to rhizobia in this country and were used for further assays.

### 3.2. High Compatibility Between Abelina and Highly Osmotic-Tolerant Rhizobia

Under severe abiotic stress conditions, legumes have a reduced number of nodules, and their nitrogen-fixing activity is suppressed [35]. Tolerance to abiotic stress in rhizobia is crucial for soybean cultivation in specific environments [35]. Many rhizobia isolated from the Abelina nodules were highly tolerant to osmotic stress (Figure 2). Abelina is a cultivar that is widely grown in Poland. In Łączka, eastern Poland, Abelina showed a higher biomass and an increased accumulation of atmospheric nitrogen compared to Merlin [36]. Despite the differences between eastern and western Poland, Abelina potentially represents a soybean that is more suited to the drought environment in Poland and has a higher nodule formation capacity. When rhizobia were isolated from German soil using the Japanese cultivar *Glycine max* Enrei and the European cultivar Merlin as host plants, the isolates from Merlin were more tolerant to low-temperature stress and better adapted to the German environment [26]. As all three soybean cultivars used in this study were European varieties, no significant differences emerged. However, the results suggest the possibility that Abelina, which shows good growth in water-deficient Polish environments, may be suitable for the isolation of rhizobia with high osmotic stress tolerance.

### 3.3. Phylogenetic Characteristics of Locally Adapted Bradyrhizobium Strains Contribute to Soybean Growth Under Abiotic Stress Conditions

*Bradyrhizobium* spp. were isolated from the soil of various fields in Poland using three soybean cultivars as hosts. As *Bradyrhizobium* rhizobia are known to have a tight symbiosis with soybeans, these locally adapted *Bradyrhizobium* strains and Polish soybean cultivars were expected to show a beneficial relationship [37]. In the MLST-based taxonomy, five strains selected from these *Bradyrhizobium* strains showed higher homology to *B*. *japonicum* USDA 123 than to *B*. *japonicum* USDA 6^T^, a major soybean rhizobium (Figure S4a). Notably, *B. japonicum* USDA 123 originates from the United States. Although this strain is a highly competitive rhizobium, it has a relatively low nitrogen-fixing capacity [38,39]. In Minnesota, it was reported that in co-inoculation with *B. japonicum* USDA 123 and *B. diazoefficiens* USDA 110^T^ for soybean, *B. japonicum* USDA 123 and the locally adapted serocluster 123 rhizobia dominated the soybean root nodules, whereas inoculation with *B. diazoefficiens* USDA 110^T^ inhibited root nodule formation [37,40]. Madrzak et al. isolated soybean rhizobia from four sites located in western Poland [41]. All isolates were obtained from soybean fields with inoculants, and 87.5% of the serologically analyzed *B*. *japonicum* isolates belonged to serocluster 123, indicating that serocluster 123 is highly competitive in Poland. However, these isolates were not serologically identified in the present study. To describe the relationship among soybeans, *Bradyrhizobium*, and the serotype of rhizobium, serotyping is required. In the eastern region of Germany, neighboring Poland, some *Bradyrhizobium* spp. were isolated from soybean cultivation fields with *Bradyrhizobium* inoculants using methods similar to those used in the present study [26]. However, the homology of the housekeeping genes (*atpD* and *recA*) in the isolates in this study was low. The five *Bradyrhizobium* strains subjected to MLST in this study were particularly stress-tolerant bacteria. Therefore, although it is possible that a closely related strain of *B. japonicum* USDA 123 was only identified by chance, it is highly likely that a closely related strain of *B. japonicum* USDA 123 was specifically adapted to the Polish environment and involved in soybean nodule formation. According to Yuan et al. (2020), the German isolates improved the growth of Merlin under cold conditions [26]. In this study, the isolates were more effective on Abelina under water-deficient conditions than on Merlin under cold conditions (Figure 5 and Figure S5). These results suggest that slight differences in the climate and environment as well as adapted varieties were reflected in these results. Our results indicate the importance of the isolation of rhizobia adapted to the cultivation conditions, the location, and climatic conditions.

Plant assays under abiotic stress conditions demonstrated that the isolated locally adapted *Bradyrhizobium* strains have the potential to improve soybean growth in western Poland. In particular, under drought stress conditions, *B*. *japonicum* PSN49 exhibited a high biomass and nitrogen content (Figure 5). The bioassay also showed that PSN49 had a high salinity stress tolerance on YMA plates (Table S2). Thus, the plant and bioassay results were consistent. These findings indicate that the tolerance of rhizobium to drought stress directly influences the effectiveness of soybean inoculation under water-deficient conditions. As mentioned above, PMN37 and PSN49 are *B*. *japonicum* USDA 123-related strains. To date, no reports have suggested that *B. japonicum* USDA 123 improves soybean growth under abiotic stresses or mitigates abiotic stress in soybeans. However, 79% of the serocluster 123 USDA rhizobia, including *B. japonicum* USDA 123, can biosynthesize polysaccharides in nodules; in contrast, *B. diazoefficiens* USDA 110^T^ and *B*. *japonicum* USDA 6^T^ do not [42]. Moreover, when temperatures are reduced to 30, 25, and 20 °C, *B. japonicum* USDA 123 up-regulates the expression of *nodC* (one of the nodule formation-associated genes) [43]. In this study, the inoculation of every soybean cultivar with PMN37 or PSN49 led to an increase in nodule dry weight (Figures S5 and S6), and the rates of increase were extremely high (50–114% increase compared to *B. diazoefficiens* USDA 110 inoculation). Shiro et al. reported that *B. japonicum* USDA 123 increased its competitiveness under cold conditions when soybeans were inoculated with other *Bradyrhizobium* strains [43]. Therefore, PMN37 and PSN49 exhibited high potential under severe environmental conditions in Poland. An increase in nodule dry weight directly induces host plant biomass under drought stress. However, there are other factors to be considered, such as cold stress tolerance.

In particular, our results indicated a very close relationship among three factors: the soybean cultivar Abelina, locally adapted *Bradyrhizobium* strains, and water-deficient stress conditions. Approximately 86% of the strains isolated from Abelina root nodules showed osmotic stress tolerance (survival on YMA containing 10% mannitol; Figure 2). Regarding the other host cultivar isolates, 58% and 69% of the strains from Merlin and Sultana, respectively, showed osmotic stress tolerance. Moreover, the locally adapted *Bradyrhizobium* strains only yielded a significantly high biomass in Abelina, whereas they only had positive effects on Merlin to some extent. PMN37 and PSN49 were isolated from the Merlin and Sultana plants, respectively (Table S2). Although not from Abelina, a combination of Abelina-derived strains and these strains may be more effective in Poland.

### 3.4. Genomic Approach for Assessment of the Abiotic Stress Adaptation of PSN49

The genetic properties of PSN49 were revealed using whole genome sequencing, predicting the plant growth-promoting potential of this strain for soybeans under stress conditions. Many genes associated with abiotic stress adaptation have been annotated on the genome. Most of its cold-inducible proteins are involved in RNA metabolism, such as the cold shock family of proteins (Csps) and the RNA helicase DeaD [44]. Low temperatures cause the stabilization of the secondary structures of nucleic acids, which can affect transcription, RNA degradation, and translation [45]. Csps are among the most up-regulated proteins in response to cold shock and are nucleic acid-binding proteins containing a highly conserved cold shock domain that harbors the nucleic acid-binding motifs [45,46]. Csps function by binding to RNA and promoting the formation of single-stranded RNA by unwinding bases [47,48]. Csps promote protein translation and have been suggested to contribute to translational selectivity at low temperatures [49]. In addition, Csps function in the regulation of the transcription of two cold shock genes, *hns* and *gyrA* [50,51]. The genome of PSN49 harbors more copies of *cspA* genes than that of *B. diazoefficiens* USDA 110^T^. According to the NCBI database, the numbers of Csp-coding genes of the other *B. japonicum* strains, USDA 6^T^, USDA 135, and SEMIA 5079, are eleven, twelve, and six, respectively. *R. etli* CFN 42, *Rhizobium leguminorasum* ATCC 14479, and *Ensifer meliloti* MABNR56 have eight, nine, and nine copies of Csps, respectively (Table S8). This suggests the specificity of the copy number of Csps in the genome of PSN49. *B. diazoefficiens* USDA 30 and *B. elkanii* USDA 31 also have more copies of cold shock proteins (Figure 6a; Table S7). These strains were isolated from the northern regions in the USA and improved the yield, leaf area, protein production, nitrogen content, and nodule formation of soybeans more than the commercial Canadian strain *B. japonicum* 532 C under field conditions in Canada [52,53]. Our results indicate a possible relationship among the following three factors: soybean growth promotion under abiotic stress conditions, the cold environment of the isolation source, and cold shock proteins. Helicase DeaD can melt secondary structures and facilitate their degradation by the cold shock exoribonucleases PNPase and RNase R [44]. DEAD-box family genes in soybean are induced by rhizobia and/or highly expressed in the nodule; thus, DEAD-box family proteins play a crucial role in soybean–rhizobium symbiosis [54]. The *dbpA* gene is one of the DEAD-box family protein-coding genes specifically identified on the genome of PSN49 (Figure 6b). In *Yersinia pseudotuberculosis*, a *Proteobacteria*, *dbpA* mutation significantly impaired growth at low temperatures, suggesting a requirement for this gene in *Y. pseudotuberculosis* under cold stress conditions [55]. In addition, under desiccation-induced stress, a DEAD-box ATP-dependent RNA helicase coding gene is induced in *B. japonicum* [56]. It has been suggested that there is a correlation between the induction of DEAD-box RNA helicases and salinity and drought stress in plants [57]. Only five strains, with the exception of PSN49, had this protein in our plotting results; therefore, it is difficult to predict the functions of this protein in *Bradyrhizobium*. It may play an important role in soybean inoculants under stress conditions.

The adaptation of bacterial cell membranes to cold and osmotic stress involves membrane fluidity through the rapid desaturation of fatty acids [44,58]. At low temperatures, this occurs through the induction of fatty acid desaturase, regulated by the sensor kinase DesK and the response regulator DesR in *Bacillus subtilis* [59]. In *Pseudomonas aeruginosa*, a mutant of fatty acid desaturase, regulation is inhibited by high concentrations of NaCl, and the expression of osmoprotectant synthesis genes is suppressed [58]. No description of the *desR* gene in *Bradyrhizobium* has been reported; therefore, it is difficult to predict the function of this gene in PSN49. However, there is a gene coding for a sensor histidine kinase that is located five genes upstream of the *desR* gene in this genome. It is possible that these genes are involved in the regulation of fatty acid desaturase.

The BLASTp plots of the annotated genes revealed the specificity of the trehalose-6-phosphate synthase in PSN49 (Figure 6b). The first gene (*otsA*_1) was predicted to be a common protein in the genus of *Bradyrhizobium*, whereas the second and third were additional copies of this protein. *B. elkanii* USDA 31 and USDA 46 possessed the second gene. *B. japonicum* USDA 5 and USDA 28, *B. diazoefficiens* USDA 30, and *B. elkanii* USDA 46 had the third gene. The *otsA* gene of *Bradyrhizobium* is involved in intracellular trehalose synthesis and accumulation induced by salinity and desiccation stresses [56,60]. Sugawara et al. showed that *otsA* is important for soybean root nodule maturation [60]. The application of trehalose improved *B. japonicum* survival on soybean seeds under desiccation stress, indicating the importance of trehalose for soybean rhizobial inoculants [61]. Trehalose concentrations are higher in *B. elkanii* USDA 46 and *B. japonicum* USDA 5 and USDA 28 on soybean seeds than that in *B. diazoefficiens* USDA 110 [62]. Streeter also found a significant positive correlation between trehalose accumulation and seed survival across strains, as well as the increased survival of *B. japonicum* USDA 5 and USDA 28. In addition, the aforementioned *B. diazoefficiens* USDA 30 and *B. elkanii* USDA 31 strains are low-temperature-tolerant rhizobia that promote soybean growth in cold environments [52,53]. These five strains and PSN49 partially share multiple copies of the trehalose-6-phosphate synthase orthologue. This result indicates a possible specificity of trehalose synthesis in PSN49 and the importance of trehalose for the relationship between *Bradyrhizobium* bacteria and abiotic stress. Therefore, these putative proteins in PSN49 are predicted to play important roles in promoting soybean growth under cold and water-deficient conditions. Interestingly, PSN49 possessed an orthologue that is common in *B. elkanii*. Soybean nodules formed by a *B. elkanii* strain accumulate higher trehalose concentrations [63]. It is difficult to understand the mechanisms by which PSN49 acquired this orthologue in the present study alone. Our results indicate that this strain is an important genetic resource, providing not only a soybean inoculant under stress conditions but also new insights into the relationship between bacterial trehalose and abiotic stress.

The PSN49 genome contains many genes associated with cold and water-deficient stress, including these three types of genes. These genes are predicted to be involved in the growth promotion of soybeans under abiotic stress conditions by PSN49. However, these whole-genome analysis results are only predictions and do not indicate the phenotypes of these strains. Further investigation is required to describe the contribution of these abiotic stress-associated genes to soybean growth.

## 4. Materials and Methods

### 4.1. Isolation of Soybean Rhizobia from Polish Soils

Nineteen soil samples were collected from different fields in western Poland (Table 1, Figure 1). The fields were divided into four regions. Seven fields (soil sample nos. 1 and 14–19) were located in Nowy Roznow/Nowe Goluszowice (50°11′ N; 17°49′ E), three (soil sample nos. 2, 4, and 5) were in the Pawlowice/śląskie voivodship (49°58′ N; 18°43′ E), one (soil sample no. 3) was in Wroclaw (51°18′ N; 17°11′ E), and eight (soil sample nos. 6–13) were in Tarnow (50°35′ N; 16°49′ E). The sampling sites were categorized into two types. Nos. 1–9 were fields with no history of soybean cultivation, and nos. 10–19 were fields with a history of soybean cultivation. All soil samples were stored at 4 °C after collection.

Three cultivars of European soybeans, namely Abelina, Merlin, and Sultana, were used as trap hosts for rhizobial isolation. The surface of the soybean seeds was sterilized by immersion in 70% ethanol for 30 s followed by 3% sodium hypochlorite for 2 min. The seeds were rinsed 4 times with sterilized distilled water and incubated at 28 °C for 2 d. The germinated seedlings were transplanted into 300 mL glass jars containing sterilized vermiculite (Yoshino Gypsum, Tokyo, Japan) mixed with 5 g of the soil sample [16]. After the jars were transferred to the indoor cultivation room, plants were cultivated at 25 °C for 4 weeks under a 16 h light/8 h dark photoperiod. A water content of 60 mL/100 g was maintained in the jars via irrigation with a sterilized nitrogen-free nutrient solution until harvest. The nitrogen-free solution was prepared as described by Artigas-Ramirez et al. (2019) [16]. After 4 weeks, the soybean plants were uprooted from the jars. The roots were washed with tap water, and the root nodules were removed. The detached nodules were surface-sterilized by soaking in 70% ethanol for 1 min and 3% sodium hypochlorite for 2 min. They were washed four times with sterilized distilled water and crushed in 300 µL of 15% glycerol. Then, 10 µL of the suspension was spread on 1.5% yeast extract mannitol agar (YMA) plates [16]. After incubation at 28 °C for 3–10 d, single colonies were selected. Pure colonies were obtained for further assays after several iterations of streaking onto fresh YMA plates. The isolates were used to further evaluate abiotic stress tolerance, genetic characteristics, and symbiotic performance.

### 4.2. Abiotic Stress Tolerance Assays

Phenotypic characteristics in response to abiotic stress were evaluated to screen for environmentally adaptable Polish strains. The tolerance of the isolated bacteria to low and high temperatures, as well as osmotic and salinity stresses, was assessed. Briefly, 10 µL of the culture was spotted on YMA plates and incubated at 4, 10, 20, 28, 35, and 40 °C for 3–14 d to test temperature stress tolerance abilities [26]. YMA supplemented with 1, 5, 10, or 15% (*w*/*v*) mannitol and 0, 1, 2, 3, or 4% (*w*/*v*) NaCl was used for the osmotic and salinity stress tolerance tests, respectively [26,64]. The strains were incubated on each plate at 28 °C for 3–14 d. Bacterial growth was evaluated, and the growth on the YMA plates served as a control. The stress tolerance of the strains was scored according to the growth rate of each colony as follows: no growth (–), 10–60% growth compared to the control (+), 60–80% growth compared to the control (++), and 80–100% growth compared to the control (+++).

### 4.3. Genomic DNA Extraction

Forty-nine strains were selected as the top-performing strains based on their origin and stress tolerance. The isolates were incubated in YM broth at 28 °C for 3–7 d. Before DNA isolation, the cells were washed with phosphate-buffered saline. Total genomic DNA was extracted from the rhizobial cells using a Wizard Genomic DNA Purification Kit (Promega, Madison, WI, USA).

### 4.4. DNA Amplification and Sequencing of 16S rRNA and Symbiosis-Associated Genes

A PCR amplification and sequencing of the 16S rRNA, nodulation protein D (*nodD*), and nitrogenase reductase (*nifH*) gene fragments from the representative 44–49 strains was performed. The 50 μL reaction mixture for PCR amplification included the following components: 2 μL primer sets (10 μM each), 25 μL Taq DNA polymerase Master Mix (GO-Taq, Promega, Madison, WI, USA), and 50–150 ng DNA template. The primer sets and PCR conditions are listed in Table S9 [13,65,66,67]. The *nifH* genes of some strains were amplified using nested PCR [67]. The 25 μL reaction mixture for nested PCR amplification included the following components: 0.5 μL primer sets (10 μM each), 12.5 μL Taq DNA polymerase Master Mix (GO-Taq, Promega, Madison, WI, USA), and 150 ng DNA template or PCR-first step production. A FastGene Gel/PCR Extraction Kit (Nippon Genetics, Tokyo, Japan) was used to purify the PCR products. Purified DNA fragments were sequenced with primer sets (Table S10) using an ABI Prism 3500 Genetic Analyzer (Applied Biosystems, Waltham, MA, USA) according to the manufacturer’s protocol.

Phylogenetic trees were constructed based on the nucleotide sequences of each strain and compared with the sequences registered in the GenBank nucleotide sequence database using BLAST (v10.2). Multiple sequences were aligned, and phylogenetic trees were established based on maximum composite likelihood analysis using the Tamura–Nei model and bootstrap method with Genetix 11 and MEGA X software (v10.2).

### 4.5. Multilocus Sequence Typing (MLST)

Based on the stress tolerance and 16S rRNA gene sequencing results, nine strains were selected for further assays in plants. These representative strains were identified and characterized based on the sequencing of four housekeeping genes, namely *atpD*, *recA*, *glnA*, and *rpoB*, and used for the phylogenetic analysis. The primer sets and PCR conditions are listed in Table S10 [68,69,70,71,72,73]. The composition of the mixture for PCR amplification and the purification of DNA fragments, as well as the methods of sequencing and identification, were the same as those used for the 16S rRNA and nitrogen fixation-associated genes. MLST was performed based on the concatenated sequences of the four genes.

### 4.6. Plant Inoculation Assay

Nine representative strains were evaluated for their symbiotic performance with soybeans in pot experiments. *B. diazoefficiens* USDA 110T was used as a positive control for the main statistical and physiological performance analyses. Here, “n.i.” (non-inoculated) served as a negative control and was treated with a sterilized nitrogen-free nutrient solution without any bacterial strain. Three European soybean cultivars (Abelina, Merlin, and Sultana) were used as trap hosts. The seeds were surface-sterilized and germinated as described above. The germinated seedlings were transplanted into 300 mL glass jars containing sterilized vermiculite (Fukushima Vermi, Fukushima, Japan), and 5 mL of each bacterial suspension (10^8^ CFU/mL) was added to each plant. The plants were grown aseptically in a growth chamber under the same conditions as those previously described for the isolation experiments. All treatments were performed in triplicate for each strain. After 4 weeks, all parts of the plants were harvested and used for measurements. Fresh roots with nodules were evaluated for nitrogenase activity using the acetylene reduction assay. The plant roots were incubated in 300 mL glass jars containing 10% acetylene gas (*v*/*v*) at 25 °C for 1 h. After incubation, the ethylene concentration in the jar was measured using a Shimadzu GC-2014 gas chromatograph (Shimadzu, Kyoto, Japan) equipped with a Porapak-N column (Agilent Technologies, Santa Clara, CA, USA). The samples were run in 1 mL for 6 min. Root nodules were removed from each root and counted. The separated shoots, roots, and nodules were dried at 80 °C for 2 d and weighed.

Additionally, pot experiments were performed to simulate the Polish environment, which included cold climates and water deficits. Two representative strains were selected based on plant assays under optimal environmental conditions. Two European soybean cultivars, Abelina and Merlin, were used as trap hosts. As a low-temperature treatment, the plants were grown at 20 °C with light for 16 h and at 10 °C in the dark for 8 h, whereas the standard conditions were maintained at 25 °C at all times. For water-deficient conditions, the plants were cultivated with half the amount of irrigation (30 mL/100 g) of the previous assays. After 6 weeks, the plants were harvested, and the acetylene reduction activity, number of nodules, and biomass were evaluated using the same method. Additionally, nitrogen content was measured using the dried powders of the plant shoots with an NC analyzer (SUMIGRAPH NCTR22, Sumika Chemical Analysis Service Ltd., Tokyo, Japan). All treatments were performed with four replicates for each strain.

### 4.7. Whole Genome Sequencing of PSN49

Whole genome sequencing of the representative *Bradyrhizobium* strain, PSN49, was performed. The genomic DNA was extracted using a Wizard Genomic DNA Purification Kit (Promega, Madison, WI, USA). After assessing the quality of the extracted DNA using a Qubit 4 fluorometer (Thermo Fisher Scientific, Waltham, MA, USA) and Femto Pulse System (Agilent, Santa Clara, CA, USA), the sequencing library was prepared via the SMRTbell Prep Kit 3.0 protocol (102-182-700, REV03 MAR2024, Pacific Biosciences, Menlo Park, CA, USA). Revio sequencing was performed using a Revio SMRT Cell tray (PacBio; 102-202-200) and Revio™ Polymerase Kit (PacBio; 102-739-100). After sequencing, bam files were generated with SMRT link v13.1.0.221970 and processed with samtools 1.21 [74]. The sequenced reads were assembled using Flye v.2.9.4 [75] with the default settings, and contigs < 1000 bp were removed using SeqKit v2.6.1 [76]. The genomic sequences were annotated with Prokka v1.14.6 (--rawproduct--mincontiglen 1000) [77] and DDBJ Fast Annotation and Submission Tool and were evaluated with QUAST v.5.0.2 [78] and the CheckM v1.1.3 lineage workflow (-r--ali--genes--tab_table) [79]. Taxonomy identification was performed using GTDB-Tk 2.3.2 [80] with the default option, using the Release214 database. Orthologous genes between the PSN49 and *Bradyrhizobium* strains were calculated based on OrthoFinder v2.5.5 [81]. A phylogenetic tree of *Bradyrhizobium* genomes was constructed using UBCG v2 [82]. A heat map for comparison among the proteins in the *Bradyrhizobium* genomes was created using the Interactive Tree of Life (iTOL) v6 [83].

### 4.8. Nucleotide Sequence Accession Numbers

DNA sequences were deposited in the DNA Data Bank of Japan (DDBJ) under accession numbers LC765466 to LC765506 and LC765508 to LC765515 for 16S rRNA sequences; LC767878 to LC767888, LC767890, and LC767891 for *recA* sequences; LC767731 to LC767742, LC767744, and LC767745 for *atpD* sequences; LC767892 to LC767903, LC767905, and LC767906 for *rpoB* sequences; LC767746 to LC767757, LC767759, and LC767760 for *glnA* sequences; LC767645 to LC767684 for *nodD* sequences; and LC767690 to LC767730 for *nifH* sequences. Whole genome sequences of PSN49 were CP187249–CP187256. Sequences of reference strains for whole genome analysis were obtained from NCBI database with the following accession numbers: GCF_037623515.1, GCF_000807315.1, GCF_001887695.1, GCF_029892585.1, GCF_021229375.1, GCF_024170805.1, GCF_024171065.1, GCF_000284375.1, GCF_024171145.1, GCF_024170955.1, GCA_024171185.1, GCF_024171225.1, GCF_024171205.1, GCF_024809375.1, GCF_023278185.1, GCF_001642675.1, GCF_000482525.1, GCF_040549625.1, and GCF_002278135.3.

### 4.9. Statistical Analyses

Data were statistically analyzed using two-tailed Dunnett’s test at *p* < 0.05 (R v4.3.1) and compared to values for *B. diazoefficiens* USDA 110^T^ as positive control.

## 5. Conclusions

In conclusion, we identified promising rhizobial candidates for soybean cultivation in Poland. *B*. *japonicum* PMN37 and PSN49 can potentially improve the growth of soybeans exposed to severe environmental conditions. These isolates are expected to acquire broad niches and adapt to the local environment with high competitiveness because they are related to *B*. *japonicum* USDA 123. For these strains to be applied in soybean production in Poland, it is necessary to address various problems related to the soil environment, climatic conditions, and increase in final yield. This study provides valuable insights by identifying locally adapted rhizobium material that can be practically applied in soybean cultivation in Poland and elucidating the genetic and historical background of the rhizobium material. We believe that this study will serve as a foundation for technological developments that will revolutionize soybean cultivation in Poland.

## Figures and Tables

**Table 1:** Soil sampling fields and their crop histories.

Soil Sample No.	Site Name	Soil Sample Collection Date	Soil Gravity Categories	Clay (%) (<0.02 mm)	Silt (%) (0.1–0.02 mm)	Sand (%) (1–0.1 mm)	Crop Rotation History (Winter–Spring–Autumn–Summer)	Soybean Harvest Date	pH
1	NOWY ROŻNÓW	15 November 2019	Light	39	45	16	Spring wheat–oilseed rape– winter wheat–maize	-	6.3
2	PAWŁOWICE	12 November 2019	Light	20	15	65	Winter oilseed rape–winter wheat– winter oilseed rape–winter wheat	-	7.9
3	WROCŁAW	14 November 2019	Medium	25	18	57	Oat–winter oilseed rape–winter wheat–winter oilseed rape–winter wheat	-	6.2
4	PAWŁOWICE	14 November 2019	Medium	23	15	62	Winter oilseed rape–winter wheat– winter oilseed rape–winter wheat	-	6.5
5	PAWŁOWICE	21 November 2019	Light	16	14	70	Oat–winter triticale–flax– winter oilseed rape–winter triticale	-	7.6
6	TARNÓW	26 November 2019	Heavy	40	45	15	Winter wheat–sugar beet– spring barley–winter oilseed rape	-	6.2
7	TARNÓW	27 November 2019	Heavy	41	48	11	Spring barley–winter oilseed rape– winter wheat–sugar beet	-	6.3
8	TARNÓW	28 November 2019	Heavy	43	42	15	Sugar beet–spring barley– winter oilseed rape–winter wheat	-	6.1
9	TARNÓW	26 November 2019	Heavy	45	41	14	Spring barley–vegetable– spring barley–vegetable	-	6.5
10	TARNÓW	20 November 2019	Heavy	47	43	10	Soybean–winter wheat– potatoes–soybean	2019	6.3
11	TARNÓW	17 November 2019	Heavy	42	47	11	Winter wheat–winter oilseed rape– winter wheat–soybean	2019	6.2
12	TARNÓW	13 November 2019	Heavy	44	42	14	Soybean–spring wheat– vegetable–winter wheat	2016	6.1
13	TARNÓW	8 November 2019	Heavy	39	46	15	Maize–winter wheat–vegetable– spring wheat–soybean–vegetable– winter wheat–vegetable	2015	6.6
14	NOWY ROŻNÓW	8 November 2019	Heavy	38	47	15	Sugar beet–winter wheat– oilseed rape–winter wheat– soybean–maize–spring wheat	2017	6.5
15	NOWY ROŻNÓW	5 November 2019	Heavy	36	51	13	Sugar beet–winter wheat– oilseed rape–winter wheat– soybean–maize–spring wheat	2016	6.4
16	NOWY ROŻNÓW	6 November 2019	Heavy	37	49	14	Sugar beet–winter wheat–oilseed rape– winter wheat–soybean	2019	6.7
17	NOWY ROŻNÓW	6 November 2019	Heavy	38	48	14	Sugar beet–winter wheat–oilseed rape– winter wheat–soybean–maize	2018	6.5
18	NOWE GOLUSZOWICE	7 November 2019	Heavy	36	41	23	Winter wheat–oilseed rape– winter wheat–soybean	2019	6.7
19	NOWY ROŻNÓW	4 November 2019	Heavy	38	42	20	Winter wheat–oilseed rape– winter wheat–soybean	2019	6.5

These soil values are based on Kabala et al., 2019 [30]; Soil Survey Division Staff, 1993 [31]. The pH method was measured as described by van Reeuwijk, 2002 [32]. Here, “-“ indicates fields without a soybean history.

**Table 2:** Poland cultivar inoculation assays for nine representative strains.

Strain	Classification Based on MLST	Whole Plant Dry Weight (g)	Shoot Dry Weight (g)	Root Dry Weight (g)	Number of Nodules	Nodule Dry Weight (g)	ARA (/Nodule DW)	ARA (/Plant DW)
Non-inoculation (negative control)		0.966 ± 0.147 *	0.628 ± 0.064 **	0.338 ± 0.022	0.00	-	0.00	0.00
USDA 110 (positive control)		1.643 ± 0.156	1.352 ± 0.077	0.291 ± 0.019	33.00 ± 4.73	0.056 ± 0.003	16.40 ± 2.26	0.57 ± 0.12
PAN18	*Bradyrhizobium japonicum*	1.684 ± 0.172	1.326 ± 0.092	0.358 ± 0.027	39.67 ± 8.41	0.094 ± 0.019	8.04 ± 1.61	0.47 ± 0.15
PMN30	*Bradyrhizobium japonicum*	1.379 ± 0.272	1.045 ± 0.121	0.334 ± 0.040	49.67 ± 9.40	0.088 ± 0.011	7.88 ± 0.15	0.50 ± 0.02
PMN35	*Bradyrhizobium japonicum*	1.604 ± 0.217	1.238 ± 0.113	0.366 ± 0.015	47.00 ± 3.46	0.116 ± 0.004 *	7.85 ± 1.76	0.58 ± 0.16
PMN37	*Bradyrhizobium japonicum*	1.749 ± 0.226	1.297 ± 0.113	0.453 ± 0.018 *	51.67 ± 3.18	0.123 ± 0.015 **	9.11 ± 2.06	0.66 ± 0.20
PSN49	*Bradyrhizobium japonicum*	1.921 ± 0.529	1.433 ± 0.219	0.489 ± 0.090 **	37.67 ± 3.33	0.108 ± 0.016	11.16 ± 3.32	0.58 ± 0.08
PAT4	*Rhizobium* sp.	1.583 ± 0.078	1.163 ± 0.060	0.420 ± 0.020	45.67 ± 7.51	0.087 ± 0.027	21.14 ± 9.83	0.85 ± 0.19
PAN13	*Rhizobium* sp.	1.620 ± 0.351	1.221 ± 0.163	0.399 ± 0.040	46.67 ± 1.67	0.103 ± 0.011	7.35 ± 1.30	0.47 ± 0.06
PMN31	*Rhizobium* sp.	1.122 ± 0.250	0.830 ± 0.118 *	0.292 ± 0.027	59.00 ± 6.03	0.089 ± 0.011	14.14 ± 3.70	1.09 ± 0.24
PAN21	*Ensifer* sp.	1.013 ± 0.145 *	0.748 ± 0.040 **	0.265 ± 0.054	75.33 ± 10.17 **	0.049 ± 0.003	12.22 ± 2.64	0.58 ± 0.09

ARA represents acetylene reduction activity, reported as µmol h^−1^ g^−1^ nodule dry weight. USDA 110 is *Bradyrhizobium diazoefficiens* USDA 110^T^. Results for each strain were compared with those for USDA 110 using Dunnett’s test; * *p* < 0.05, ** *p* < 0.01.

**Table 3:** Overview of the PSN49 genome.

Strain	*Bradyrhizobium japonicum* PSN49	*Bradyrhizobium japonicum* USDA 123	*Bradyrhizobium japonicum* USDA 6^T^	*Bradyrhizobium diazoefficiens* USDA 110^T^
Total length	11,082,922	10,457,665	9,207,384	9,106,064
GC (%)	63.23	63.27	63.67	64.06
CDS	10,931	10,253	8421	8586
rRNA	6	3	6	3
tRNA	61	59	58	55
Accession number	CP187249–CP187256	GCA_000482525.1	GCA_000284375.1	GCA_001642675.1
OrthoANI value (%) to USDA 123	99.77	-	-	-
OrthoANI value (%) to USDA 6	95.46	95.40	-	-
OrthoANI value (%) to USDA 110	89.53	89.40	90.15	-

## Data Availability

The datasets generated and/or analyzed during the current study are available from the corresponding author upon reasonable request.

## Supplementary Materials

The following supporting information can be downloaded at https://www.mdpi.com/article/10.3390/plants14121786/s1, Figure S1: Rhizobia isolation by using Merlin cultivation as a host trap; Figure S2: Phylogenetic tree based on *nodD* gene sequences of the representative strains; Figure S3: Phylogenetic tree based on the *nifH* gene sequences of the representative strains; Figure S4: Phylogenetic trees based on the MLST of the promising inoculants; Figure S5: Plant inoculation assay under the cold condition (Merlin); Figure S6: Plant inoculation assay under the water deficient condition (Merlin); Table S1: Number of isolates from Polish soils; Table S2: Phenotypic characterization and 16S rRNA gene identification of 49 representative strains; Table S3: Merlin cultivar inoculation assay for the representative nine strains; Table S4: Sultana cultivar inoculation assay for the representative nine strains; Table S5: Plant inoculation assay for PSN49 under the abiotic stress conditions in Abelina; Table S6: Contigs of PSN49 genome; Table S7: Cold and drought stress-associated genes annotated on the genomes of PSN49 and reference strains; Table S8: Overview and number of copies of Csps on reference rhizobial strains; Table S9: Symbiotic primer sets and PCR condition analysis; Table S10: Primer sets and PCR conditions for identification and MLST.
