# Supplementary material for: Genetic Characterization and Symbiotic Performance of Soybean Rhizobia Under Cold and Water-Deficient Conditions in Poland
Source: Plants (Basel). 2025 Jun 11;14(12):1786. doi: 10.3390/plants14121786 (PMC12197054; doi:10.3390/plants14121786)
Supplement: Supplementary file 1 [file plants-14-01786-s001.zip › plants-3651416-supplementary.pptx]

## Slide 1
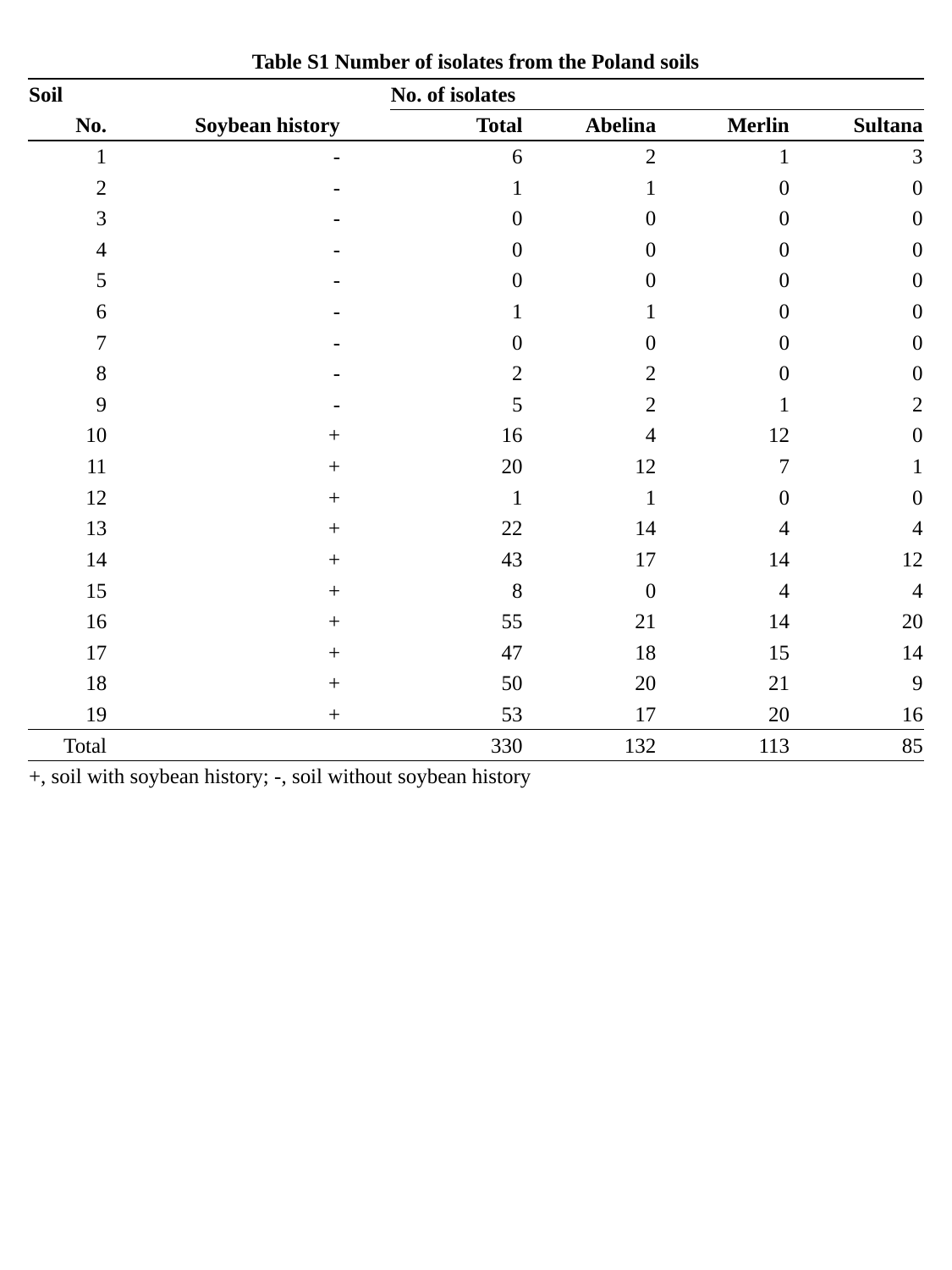

| Table S1 Number of isolates from the Poland soils | | | | | | |
| --- | --- | --- | --- | --- | --- | --- |
| Soil | | | No. of isolates | | | |
| No. | Soybean history | | Total | Abelina | Merlin | Sultana |
| 1 | - | | 6 | 2 | 1 | 3 |
| 2 | - | | 1 | 1 | 0 | 0 |
| 3 | - | | 0 | 0 | 0 | 0 |
| 4 | - | | 0 | 0 | 0 | 0 |
| 5 | - | | 0 | 0 | 0 | 0 |
| 6 | - | | 1 | 1 | 0 | 0 |
| 7 | - | | 0 | 0 | 0 | 0 |
| 8 | - | | 2 | 2 | 0 | 0 |
| 9 | - | | 5 | 2 | 1 | 2 |
| 10 | + | | 16 | 4 | 12 | 0 |
| 11 | + | | 20 | 12 | 7 | 1 |
| 12 | + | | 1 | 1 | 0 | 0 |
| 13 | + | | 22 | 14 | 4 | 4 |
| 14 | + | | 43 | 17 | 14 | 12 |
| 15 | + | | 8 | 0 | 4 | 4 |
| 16 | + | | 55 | 21 | 14 | 20 |
| 17 | + | | 47 | 18 | 15 | 14 |
| 18 | + | | 50 | 20 | 21 | 9 |
| 19 | + | | 53 | 17 | 20 | 16 |
| Total | | | 330 | 132 | 113 | 85 |
| +, soil with soybean history; -, soil without soybean history | | | | | | |

## Slide 2
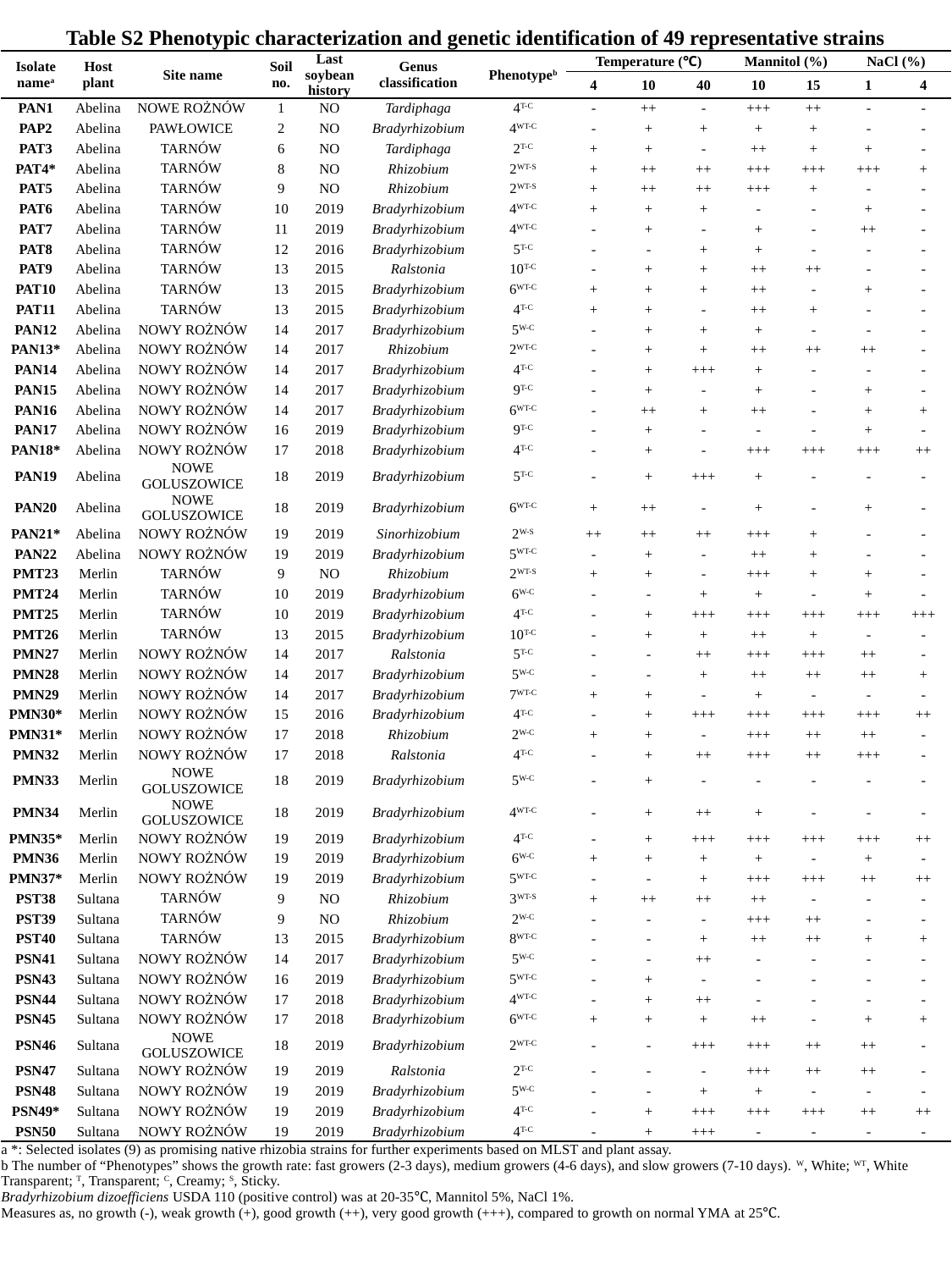

| Table S2 Phenotypic characterization and genetic identification of 49 representative strains | | | | | | | | | | | | | |
| --- | --- | --- | --- | --- | --- | --- | --- | --- | --- | --- | --- | --- | --- |
| Isolate namea | Host plant | Site name | Soil no. | Last soybean history | Genus classification | Phenotypeb | Temperature (℃) | | | Mannitol (%) | | NaCl (%) | |
| | | | | | | | 4 | 10 | 40 | 10 | 15 | 1 | 4 |
| PAN1 | Abelina | NOWE ROŻNÓW | 1 | NO | Tardiphaga | 4T-C | - | ++ | - | +++ | ++ | - | - |
| PAP2 | Abelina | PAWŁOWICE | 2 | NO | Bradyrhizobium | 4WT-C | - | + | + | + | + | - | - |
| PAT3 | Abelina | TARNÓW | 6 | NO | Tardiphaga | 2T-C | + | + | - | ++ | + | + | - |
| PAT4\* | Abelina | TARNÓW | 8 | NO | Rhizobium | 2WT-S | + | ++ | ++ | +++ | +++ | +++ | + |
| PAT5 | Abelina | TARNÓW | 9 | NO | Rhizobium | 2WT-S | + | ++ | ++ | +++ | + | - | - |
| PAT6 | Abelina | TARNÓW | 10 | 2019 | Bradyrhizobium | 4WT-C | + | + | + | - | - | + | - |
| PAT7 | Abelina | TARNÓW | 11 | 2019 | Bradyrhizobium | 4WT-C | - | + | - | + | - | ++ | - |
| PAT8 | Abelina | TARNÓW | 12 | 2016 | Bradyrhizobium | 5T-C | - | - | + | + | - | - | - |
| PAT9 | Abelina | TARNÓW | 13 | 2015 | Ralstonia | 10T-C | - | + | + | ++ | ++ | - | - |
| PAT10 | Abelina | TARNÓW | 13 | 2015 | Bradyrhizobium | 6WT-C | + | + | + | ++ | - | + | - |
| PAT11 | Abelina | TARNÓW | 13 | 2015 | Bradyrhizobium | 4T-C | + | + | - | ++ | + | - | - |
| PAN12 | Abelina | NOWY ROŻNÓW | 14 | 2017 | Bradyrhizobium | 5W-C | - | + | + | + | - | - | - |
| PAN13\* | Abelina | NOWY ROŻNÓW | 14 | 2017 | Rhizobium | 2WT-C | - | + | + | ++ | ++ | ++ | - |
| PAN14 | Abelina | NOWY ROŻNÓW | 14 | 2017 | Bradyrhizobium | 4T-C | - | + | +++ | + | - | - | - |
| PAN15 | Abelina | NOWY ROŻNÓW | 14 | 2017 | Bradyrhizobium | 9T-C | - | + | - | + | - | + | - |
| PAN16 | Abelina | NOWY ROŻNÓW | 14 | 2017 | Bradyrhizobium | 6WT-C | - | ++ | + | ++ | - | + | + |
| PAN17 | Abelina | NOWY ROŻNÓW | 16 | 2019 | Bradyrhizobium | 9T-C | - | + | - | - | - | + | - |
| PAN18\* | Abelina | NOWY ROŻNÓW | 17 | 2018 | Bradyrhizobium | 4T-C | - | + | - | +++ | +++ | +++ | ++ |
| PAN19 | Abelina | NOWE GOLUSZOWICE | 18 | 2019 | Bradyrhizobium | 5T-C | - | + | +++ | + | - | - | - |
| PAN20 | Abelina | NOWE GOLUSZOWICE | 18 | 2019 | Bradyrhizobium | 6WT-C | + | ++ | - | + | - | + | - |
| PAN21\* | Abelina | NOWY ROŻNÓW | 19 | 2019 | Sinorhizobium | 2W-S | ++ | ++ | ++ | +++ | + | - | - |
| PAN22 | Abelina | NOWY ROŻNÓW | 19 | 2019 | Bradyrhizobium | 5WT-C | - | + | - | ++ | + | - | - |
| PMT23 | Merlin | TARNÓW | 9 | NO | Rhizobium | 2WT-S | + | + | - | +++ | + | + | - |
| PMT24 | Merlin | TARNÓW | 10 | 2019 | Bradyrhizobium | 6W-C | - | - | + | + | - | + | - |
| PMT25 | Merlin | TARNÓW | 10 | 2019 | Bradyrhizobium | 4T-C | - | + | +++ | +++ | +++ | +++ | +++ |
| PMT26 | Merlin | TARNÓW | 13 | 2015 | Bradyrhizobium | 10T-C | - | + | + | ++ | + | - | - |
| PMN27 | Merlin | NOWY ROŻNÓW | 14 | 2017 | Ralstonia | 5T-C | - | - | ++ | +++ | +++ | ++ | - |
| PMN28 | Merlin | NOWY ROŻNÓW | 14 | 2017 | Bradyrhizobium | 5W-C | - | - | + | ++ | ++ | ++ | + |
| PMN29 | Merlin | NOWY ROŻNÓW | 14 | 2017 | Bradyrhizobium | 7WT-C | + | + | - | + | - | - | - |
| PMN30\* | Merlin | NOWY ROŻNÓW | 15 | 2016 | Bradyrhizobium | 4T-C | - | + | +++ | +++ | +++ | +++ | ++ |
| PMN31\* | Merlin | NOWY ROŻNÓW | 17 | 2018 | Rhizobium | 2W-C | + | + | - | +++ | ++ | ++ | - |
| PMN32 | Merlin | NOWY ROŻNÓW | 17 | 2018 | Ralstonia | 4T-C | - | + | ++ | +++ | ++ | +++ | - |
| PMN33 | Merlin | NOWE GOLUSZOWICE | 18 | 2019 | Bradyrhizobium | 5W-C | - | + | - | - | - | - | - |
| PMN34 | Merlin | NOWE GOLUSZOWICE | 18 | 2019 | Bradyrhizobium | 4WT-C | - | + | ++ | + | - | - | - |
| PMN35\* | Merlin | NOWY ROŻNÓW | 19 | 2019 | Bradyrhizobium | 4T-C | - | + | +++ | +++ | +++ | +++ | ++ |
| PMN36 | Merlin | NOWY ROŻNÓW | 19 | 2019 | Bradyrhizobium | 6W-C | + | + | + | + | - | + | - |
| PMN37\* | Merlin | NOWY ROŻNÓW | 19 | 2019 | Bradyrhizobium | 5WT-C | - | - | + | +++ | +++ | ++ | ++ |
| PST38 | Sultana | TARNÓW | 9 | NO | Rhizobium | 3WT-S | + | ++ | ++ | ++ | - | - | - |
| PST39 | Sultana | TARNÓW | 9 | NO | Rhizobium | 2W-C | - | - | - | +++ | ++ | - | - |
| PST40 | Sultana | TARNÓW | 13 | 2015 | Bradyrhizobium | 8WT-C | - | - | + | ++ | ++ | + | + |
| PSN41 | Sultana | NOWY ROŻNÓW | 14 | 2017 | Bradyrhizobium | 5W-C | - | - | ++ | - | - | - | - |
| PSN43 | Sultana | NOWY ROŻNÓW | 16 | 2019 | Bradyrhizobium | 5WT-C | - | + | - | - | - | - | - |
| PSN44 | Sultana | NOWY ROŻNÓW | 17 | 2018 | Bradyrhizobium | 4WT-C | - | + | ++ | - | - | - | - |
| PSN45 | Sultana | NOWY ROŻNÓW | 17 | 2018 | Bradyrhizobium | 6WT-C | + | + | + | ++ | - | + | + |
| PSN46 | Sultana | NOWE GOLUSZOWICE | 18 | 2019 | Bradyrhizobium | 2WT-C | - | - | +++ | +++ | ++ | ++ | - |
| PSN47 | Sultana | NOWY ROŻNÓW | 19 | 2019 | Ralstonia | 2T-C | - | - | - | +++ | ++ | ++ | - |
| PSN48 | Sultana | NOWY ROŻNÓW | 19 | 2019 | Bradyrhizobium | 5W-C | - | - | + | + | - | - | - |
| PSN49\* | Sultana | NOWY ROŻNÓW | 19 | 2019 | Bradyrhizobium | 4T-C | - | + | +++ | +++ | +++ | ++ | ++ |
| PSN50 | Sultana | NOWY ROŻNÓW | 19 | 2019 | Bradyrhizobium | 4T-C | - | + | +++ | - | - | - | - |
| a \*: Selected isolates (9) as promising native rhizobia strains for further experiments based on MLST and plant assay. | | | | | | | | | | | | | |
| b The number of “Phenotypes” shows the growth rate: fast growers (2-3 days), medium growers (4-6 days), and slow growers (7-10 days). W, White; WT, White Transparent; T, Transparent; C, Creamy; S, Sticky. | | | | | | | | | | | | | |
| Bradyrhizobium dizoefficiens USDA 110 (positive control) was at 20-35℃, Mannitol 5%, NaCl 1%. | | | | | | | | | | | | | |
| Measures as, no growth (-), weak growth (+), good growth (++), very good growth (+++), compared to growth on normal YMA at 25℃. | | | | | | | | | | | | | |

## Slide 3
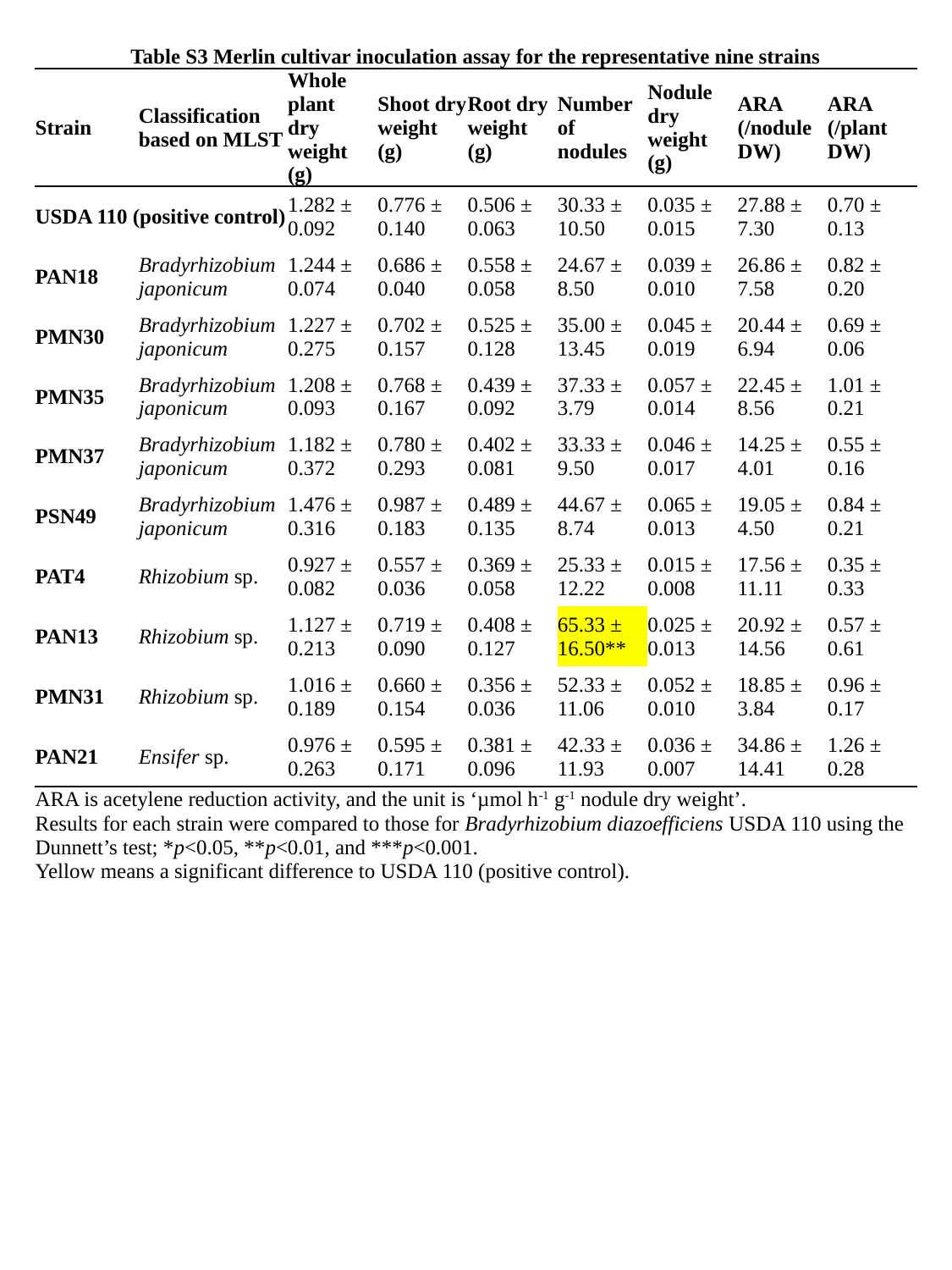

| Table S3 Merlin cultivar inoculation assay for the representative nine strains | | | | | | | | |
| --- | --- | --- | --- | --- | --- | --- | --- | --- |
| Strain | Classification based on MLST | Whole plant dry weight (g) | Shoot dry weight (g) | Root dry weight (g) | Number of nodules | Nodule dry weight (g) | ARA (/nodule DW) | ARA (/plant DW) |
| USDA 110 (positive control) | | 1.282 ± 0.092 | 0.776 ± 0.140 | 0.506 ± 0.063 | 30.33 ± 10.50 | 0.035 ± 0.015 | 27.88 ± 7.30 | 0.70 ± 0.13 |
| PAN18 | Bradyrhizobium japonicum | 1.244 ± 0.074 | 0.686 ± 0.040 | 0.558 ± 0.058 | 24.67 ± 8.50 | 0.039 ± 0.010 | 26.86 ± 7.58 | 0.82 ± 0.20 |
| PMN30 | Bradyrhizobium japonicum | 1.227 ± 0.275 | 0.702 ± 0.157 | 0.525 ± 0.128 | 35.00 ± 13.45 | 0.045 ± 0.019 | 20.44 ± 6.94 | 0.69 ± 0.06 |
| PMN35 | Bradyrhizobium japonicum | 1.208 ± 0.093 | 0.768 ± 0.167 | 0.439 ± 0.092 | 37.33 ± 3.79 | 0.057 ± 0.014 | 22.45 ± 8.56 | 1.01 ± 0.21 |
| PMN37 | Bradyrhizobium japonicum | 1.182 ± 0.372 | 0.780 ± 0.293 | 0.402 ± 0.081 | 33.33 ± 9.50 | 0.046 ± 0.017 | 14.25 ± 4.01 | 0.55 ± 0.16 |
| PSN49 | Bradyrhizobium japonicum | 1.476 ± 0.316 | 0.987 ± 0.183 | 0.489 ± 0.135 | 44.67 ± 8.74 | 0.065 ± 0.013 | 19.05 ± 4.50 | 0.84 ± 0.21 |
| PAT4 | Rhizobium sp. | 0.927 ± 0.082 | 0.557 ± 0.036 | 0.369 ± 0.058 | 25.33 ± 12.22 | 0.015 ± 0.008 | 17.56 ± 11.11 | 0.35 ± 0.33 |
| PAN13 | Rhizobium sp. | 1.127 ± 0.213 | 0.719 ± 0.090 | 0.408 ± 0.127 | 65.33 ± 16.50\*\* | 0.025 ± 0.013 | 20.92 ± 14.56 | 0.57 ± 0.61 |
| PMN31 | Rhizobium sp. | 1.016 ± 0.189 | 0.660 ± 0.154 | 0.356 ± 0.036 | 52.33 ± 11.06 | 0.052 ± 0.010 | 18.85 ± 3.84 | 0.96 ± 0.17 |
| PAN21 | Ensifer sp. | 0.976 ± 0.263 | 0.595 ± 0.171 | 0.381 ± 0.096 | 42.33 ± 11.93 | 0.036 ± 0.007 | 34.86 ± 14.41 | 1.26 ± 0.28 |
| ARA is acetylene reduction activity, and the unit is ‘µmol h-1 g-1 nodule dry weight’. | | | | | | | | |
| Results for each strain were compared to those for Bradyrhizobium diazoefficiens USDA 110 using the Dunnett’s test; \*p<0.05, \*\*p<0.01, and \*\*\*p<0.001. | | | | | | | | |
| Yellow means a significant difference to USDA 110 (positive control). | | | | | | | | |

## Slide 4
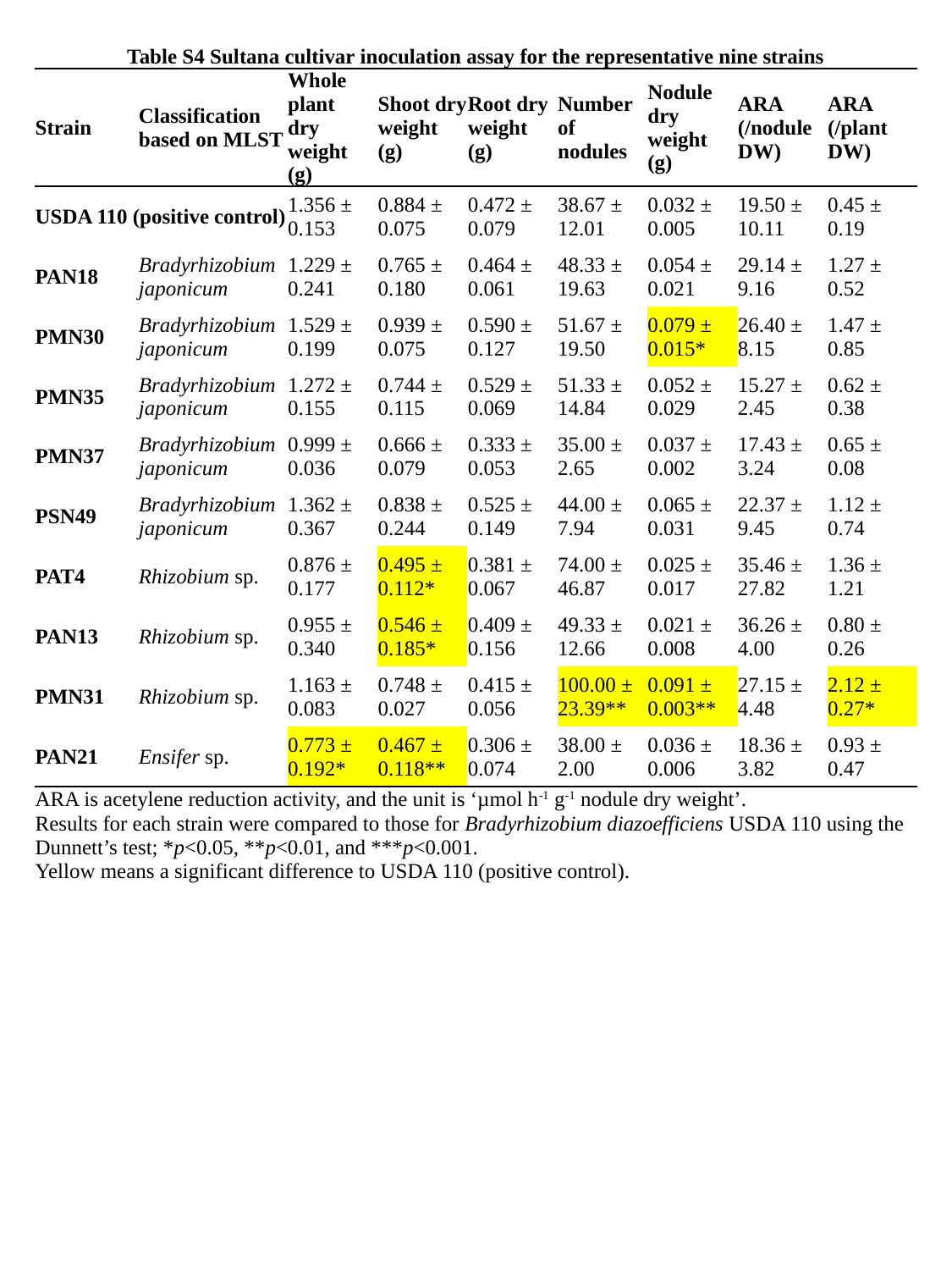

| Table S4 Sultana cultivar inoculation assay for the representative nine strains | | | | | | | | |
| --- | --- | --- | --- | --- | --- | --- | --- | --- |
| Strain | Classification based on MLST | Whole plant dry weight (g) | Shoot dry weight (g) | Root dry weight (g) | Number of nodules | Nodule dry weight (g) | ARA (/nodule DW) | ARA (/plant DW) |
| USDA 110 (positive control) | | 1.356 ± 0.153 | 0.884 ± 0.075 | 0.472 ± 0.079 | 38.67 ± 12.01 | 0.032 ± 0.005 | 19.50 ± 10.11 | 0.45 ± 0.19 |
| PAN18 | Bradyrhizobium japonicum | 1.229 ± 0.241 | 0.765 ± 0.180 | 0.464 ± 0.061 | 48.33 ± 19.63 | 0.054 ± 0.021 | 29.14 ± 9.16 | 1.27 ± 0.52 |
| PMN30 | Bradyrhizobium japonicum | 1.529 ± 0.199 | 0.939 ± 0.075 | 0.590 ± 0.127 | 51.67 ± 19.50 | 0.079 ± 0.015\* | 26.40 ± 8.15 | 1.47 ± 0.85 |
| PMN35 | Bradyrhizobium japonicum | 1.272 ± 0.155 | 0.744 ± 0.115 | 0.529 ± 0.069 | 51.33 ± 14.84 | 0.052 ± 0.029 | 15.27 ± 2.45 | 0.62 ± 0.38 |
| PMN37 | Bradyrhizobium japonicum | 0.999 ± 0.036 | 0.666 ± 0.079 | 0.333 ± 0.053 | 35.00 ± 2.65 | 0.037 ± 0.002 | 17.43 ± 3.24 | 0.65 ± 0.08 |
| PSN49 | Bradyrhizobium japonicum | 1.362 ± 0.367 | 0.838 ± 0.244 | 0.525 ± 0.149 | 44.00 ± 7.94 | 0.065 ± 0.031 | 22.37 ± 9.45 | 1.12 ± 0.74 |
| PAT4 | Rhizobium sp. | 0.876 ± 0.177 | 0.495 ± 0.112\* | 0.381 ± 0.067 | 74.00 ± 46.87 | 0.025 ± 0.017 | 35.46 ± 27.82 | 1.36 ± 1.21 |
| PAN13 | Rhizobium sp. | 0.955 ± 0.340 | 0.546 ± 0.185\* | 0.409 ± 0.156 | 49.33 ± 12.66 | 0.021 ± 0.008 | 36.26 ± 4.00 | 0.80 ± 0.26 |
| PMN31 | Rhizobium sp. | 1.163 ± 0.083 | 0.748 ± 0.027 | 0.415 ± 0.056 | 100.00 ± 23.39\*\* | 0.091 ± 0.003\*\* | 27.15 ± 4.48 | 2.12 ± 0.27\* |
| PAN21 | Ensifer sp. | 0.773 ± 0.192\* | 0.467 ± 0.118\*\* | 0.306 ± 0.074 | 38.00 ± 2.00 | 0.036 ± 0.006 | 18.36 ± 3.82 | 0.93 ± 0.47 |
| ARA is acetylene reduction activity, and the unit is ‘µmol h-1 g-1 nodule dry weight’. | | | | | | | | |
| Results for each strain were compared to those for Bradyrhizobium diazoefficiens USDA 110 using the Dunnett’s test; \*p<0.05, \*\*p<0.01, and \*\*\*p<0.001. | | | | | | | | |
| Yellow means a significant difference to USDA 110 (positive control). | | | | | | | | |

## Slide 5
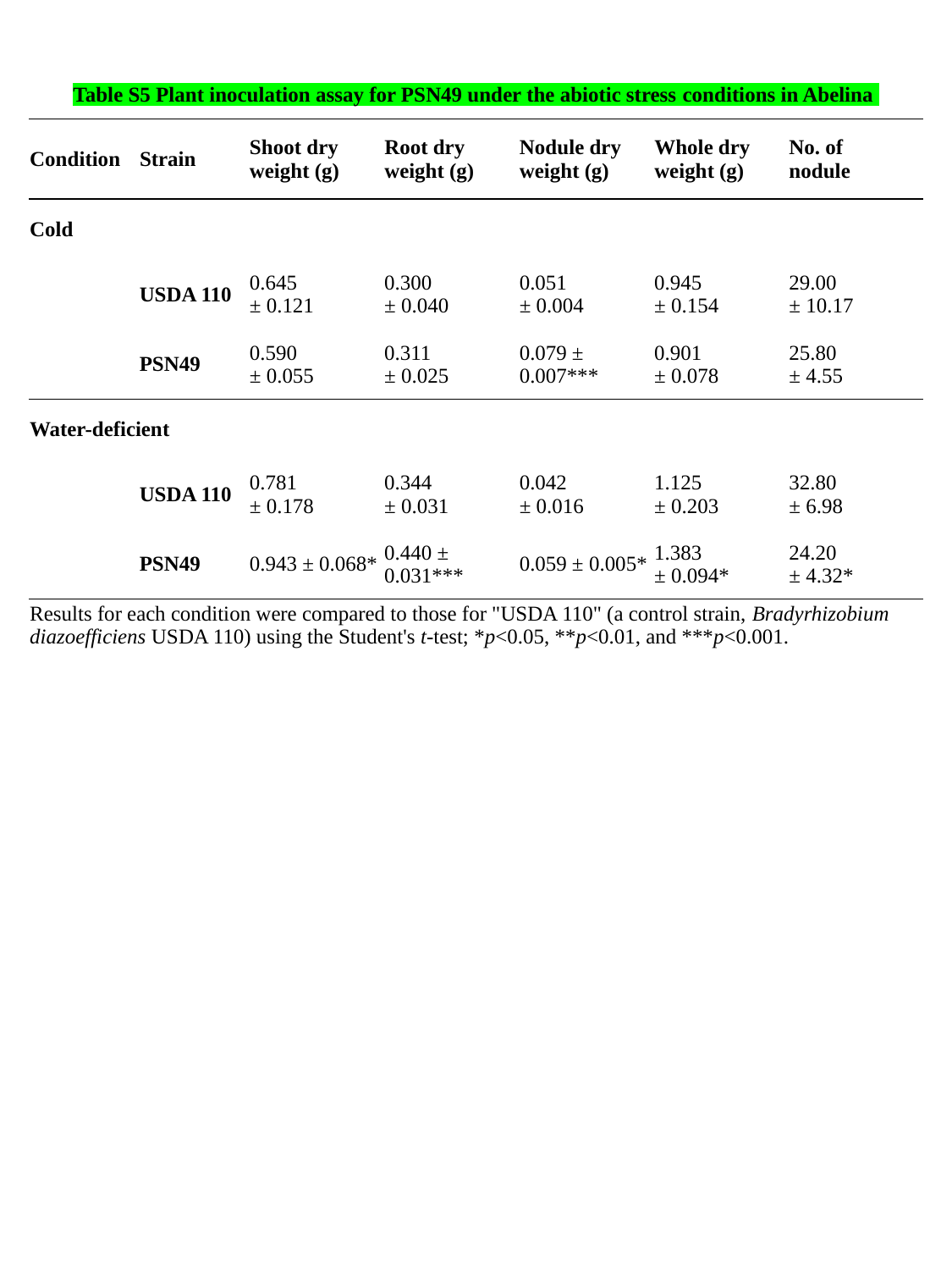

| Table S5 Plant inoculation assay for PSN49 under the abiotic stress conditions in Abelina | | | | | | |
| --- | --- | --- | --- | --- | --- | --- |
| Condition | Strain | Shoot dry weight (g) | Root dry weight (g) | Nodule dry weight (g) | Whole dry weight (g) | No. of nodule |
| Cold | | | | | | |
| | USDA 110 | 0.645 ± 0.121 | 0.300 ± 0.040 | 0.051 ± 0.004 | 0.945 ± 0.154 | 29.00 ± 10.17 |
| | PSN49 | 0.590 ± 0.055 | 0.311 ± 0.025 | 0.079 ± 0.007\*\*\* | 0.901 ± 0.078 | 25.80 ± 4.55 |
| Water-deficient | | | | | | |
| | USDA 110 | 0.781 ± 0.178 | 0.344 ± 0.031 | 0.042 ± 0.016 | 1.125 ± 0.203 | 32.80 ± 6.98 |
| | PSN49 | 0.943 ± 0.068\* | 0.440 ± 0.031\*\*\* | 0.059 ± 0.005\* | 1.383 ± 0.094\* | 24.20 ± 4.32\* |
| Results for each condition were compared to those for "USDA 110" (a control strain, Bradyrhizobium diazoefficiens USDA 110) using the Student's t-test; \*p<0.05, \*\*p<0.01, and \*\*\*p<0.001. | | | | | | |

## Slide 6
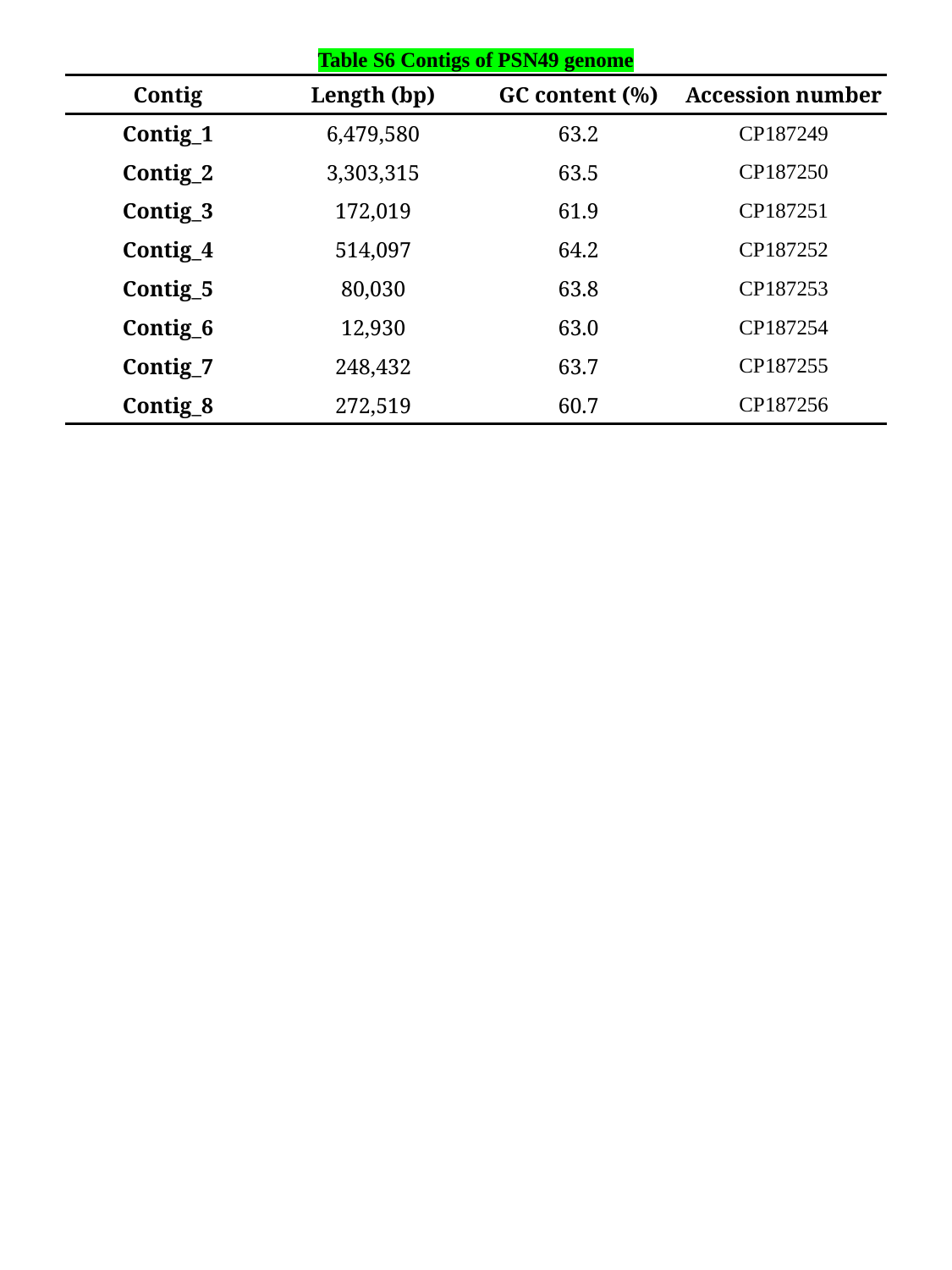

| Table S6 Contigs of PSN49 genome | | | |
| --- | --- | --- | --- |
| Contig | Length (bp) | GC content (%) | Accession number |
| Contig\_1 | 6,479,580 | 63.2 | CP187249 |
| Contig\_2 | 3,303,315 | 63.5 | CP187250 |
| Contig\_3 | 172,019 | 61.9 | CP187251 |
| Contig\_4 | 514,097 | 64.2 | CP187252 |
| Contig\_5 | 80,030 | 63.8 | CP187253 |
| Contig\_6 | 12,930 | 63.0 | CP187254 |
| Contig\_7 | 248,432 | 63.7 | CP187255 |
| Contig\_8 | 272,519 | 60.7 | CP187256 |

## Slide 7
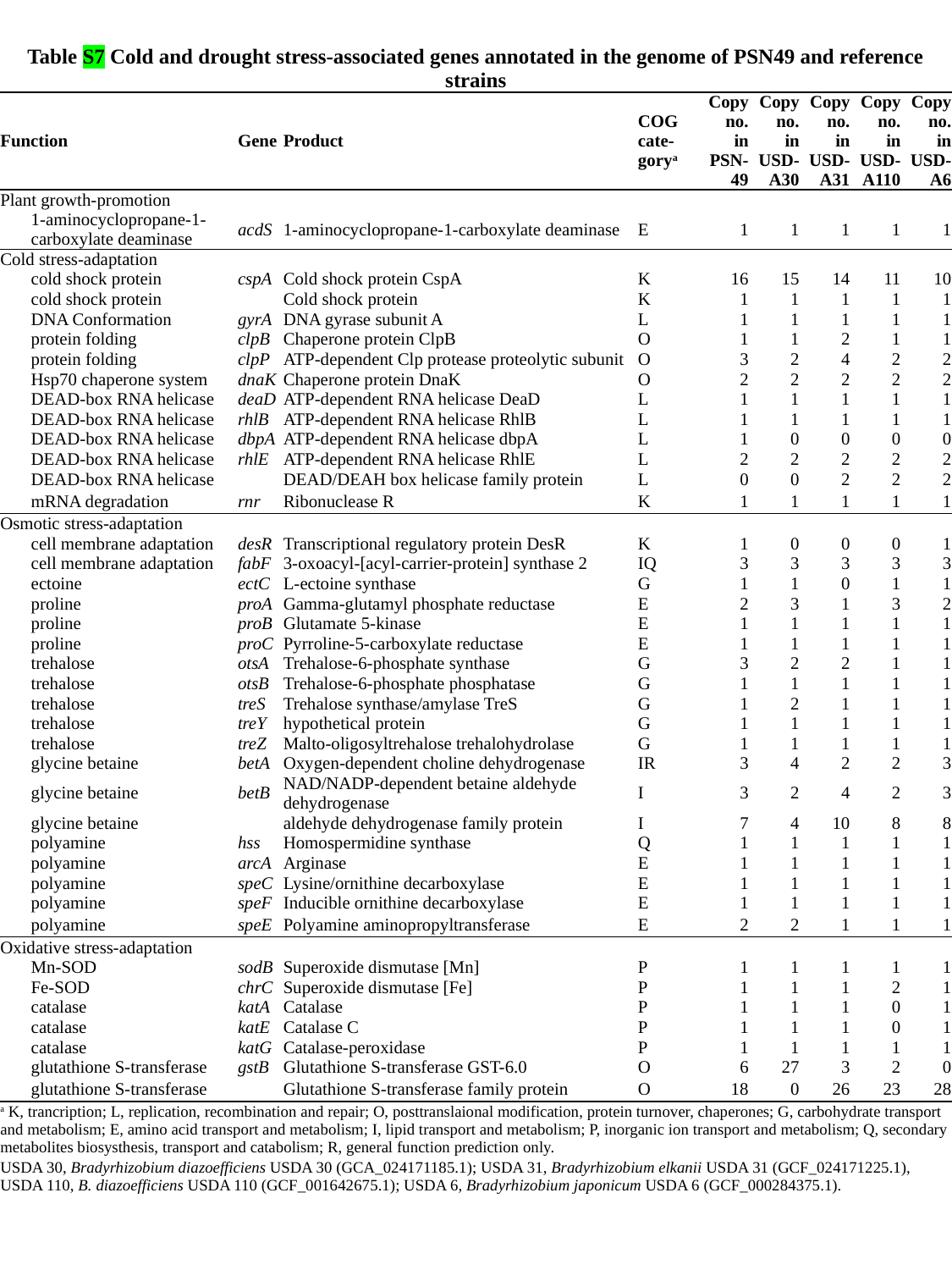

| Table S7 Cold and drought stress-associated genes annotated in the genome of PSN49 and reference strains | | | | | | | | | |
| --- | --- | --- | --- | --- | --- | --- | --- | --- | --- |
| Function | | Gene | Product | COG cate-gorya | Copy no. in PSN-49 | Copy no. in USD-A30 | Copy no. in USD-A31 | Copy no. in USD-A110 | Copy no. in USD-A6 |
| Plant growth-promotion | | | | | | | | | |
| | 1-aminocyclopropane-1-carboxylate deaminase | acdS | 1-aminocyclopropane-1-carboxylate deaminase | E | 1 | 1 | 1 | 1 | 1 |
| Cold stress-adaptation | | | | | | | | | |
| | cold shock protein | cspA | Cold shock protein CspA | K | 16 | 15 | 14 | 11 | 10 |
| | cold shock protein | | Cold shock protein | K | 1 | 1 | 1 | 1 | 1 |
| | DNA Conformation | gyrA | DNA gyrase subunit A | L | 1 | 1 | 1 | 1 | 1 |
| | protein folding | clpB | Chaperone protein ClpB | O | 1 | 1 | 2 | 1 | 1 |
| | protein folding | clpP | ATP-dependent Clp protease proteolytic subunit | O | 3 | 2 | 4 | 2 | 2 |
| | Hsp70 chaperone system | dnaK | Chaperone protein DnaK | O | 2 | 2 | 2 | 2 | 2 |
| | DEAD-box RNA helicase | deaD | ATP-dependent RNA helicase DeaD | L | 1 | 1 | 1 | 1 | 1 |
| | DEAD-box RNA helicase | rhlB | ATP-dependent RNA helicase RhlB | L | 1 | 1 | 1 | 1 | 1 |
| | DEAD-box RNA helicase | dbpA | ATP-dependent RNA helicase dbpA | L | 1 | 0 | 0 | 0 | 0 |
| | DEAD-box RNA helicase | rhlE | ATP-dependent RNA helicase RhlE | L | 2 | 2 | 2 | 2 | 2 |
| | DEAD-box RNA helicase | | DEAD/DEAH box helicase family protein | L | 0 | 0 | 2 | 2 | 2 |
| | mRNA degradation | rnr | Ribonuclease R | K | 1 | 1 | 1 | 1 | 1 |
| Osmotic stress-adaptation | | | | | | | | | |
| | cell membrane adaptation | desR | Transcriptional regulatory protein DesR | K | 1 | 0 | 0 | 0 | 1 |
| | cell membrane adaptation | fabF | 3-oxoacyl-[acyl-carrier-protein] synthase 2 | IQ | 3 | 3 | 3 | 3 | 3 |
| | ectoine | ectC | L-ectoine synthase | G | 1 | 1 | 0 | 1 | 1 |
| | proline | proA | Gamma-glutamyl phosphate reductase | E | 2 | 3 | 1 | 3 | 2 |
| | proline | proB | Glutamate 5-kinase | E | 1 | 1 | 1 | 1 | 1 |
| | proline | proC | Pyrroline-5-carboxylate reductase | E | 1 | 1 | 1 | 1 | 1 |
| | trehalose | otsA | Trehalose-6-phosphate synthase | G | 3 | 2 | 2 | 1 | 1 |
| | trehalose | otsB | Trehalose-6-phosphate phosphatase | G | 1 | 1 | 1 | 1 | 1 |
| | trehalose | treS | Trehalose synthase/amylase TreS | G | 1 | 2 | 1 | 1 | 1 |
| | trehalose | treY | hypothetical protein | G | 1 | 1 | 1 | 1 | 1 |
| | trehalose | treZ | Malto-oligosyltrehalose trehalohydrolase | G | 1 | 1 | 1 | 1 | 1 |
| | glycine betaine | betA | Oxygen-dependent choline dehydrogenase | IR | 3 | 4 | 2 | 2 | 3 |
| | glycine betaine | betB | NAD/NADP-dependent betaine aldehyde dehydrogenase | I | 3 | 2 | 4 | 2 | 3 |
| | glycine betaine | | aldehyde dehydrogenase family protein | I | 7 | 4 | 10 | 8 | 8 |
| | polyamine | hss | Homospermidine synthase | Q | 1 | 1 | 1 | 1 | 1 |
| | polyamine | arcA | Arginase | E | 1 | 1 | 1 | 1 | 1 |
| | polyamine | speC | Lysine/ornithine decarboxylase | E | 1 | 1 | 1 | 1 | 1 |
| | polyamine | speF | Inducible ornithine decarboxylase | E | 1 | 1 | 1 | 1 | 1 |
| | polyamine | speE | Polyamine aminopropyltransferase | E | 2 | 2 | 1 | 1 | 1 |
| Oxidative stress-adaptation | | | | | | | | | |
| | Mn-SOD | sodB | Superoxide dismutase [Mn] | P | 1 | 1 | 1 | 1 | 1 |
| | Fe-SOD | chrC | Superoxide dismutase [Fe] | P | 1 | 1 | 1 | 2 | 1 |
| | catalase | katA | Catalase | P | 1 | 1 | 1 | 0 | 1 |
| | catalase | katE | Catalase C | P | 1 | 1 | 1 | 0 | 1 |
| | catalase | katG | Catalase-peroxidase | P | 1 | 1 | 1 | 1 | 1 |
| | glutathione S-transferase | gstB | Glutathione S-transferase GST-6.0 | O | 6 | 27 | 3 | 2 | 0 |
| | glutathione S-transferase | | Glutathione S-transferase family protein | O | 18 | 0 | 26 | 23 | 28 |
| a K, trancription; L, replication, recombination and repair; O, posttranslaional modification, protein turnover, chaperones; G, carbohydrate transport and metabolism; E, amino acid transport and metabolism; I, lipid transport and metabolism; P, inorganic ion transport and metabolism; Q, secondary metabolites biosysthesis, transport and catabolism; R, general function prediction only. | | | | | | | | | |
| USDA 30, Bradyrhizobium diazoefficiens USDA 30 (GCA\_024171185.1); USDA 31, Bradyrhizobium elkanii USDA 31 (GCF\_024171225.1), USDA 110, B. diazoefficiens USDA 110 (GCF\_001642675.1); USDA 6, Bradyrhizobium japonicum USDA 6 (GCF\_000284375.1). | | | | | | | | | |

## Slide 8
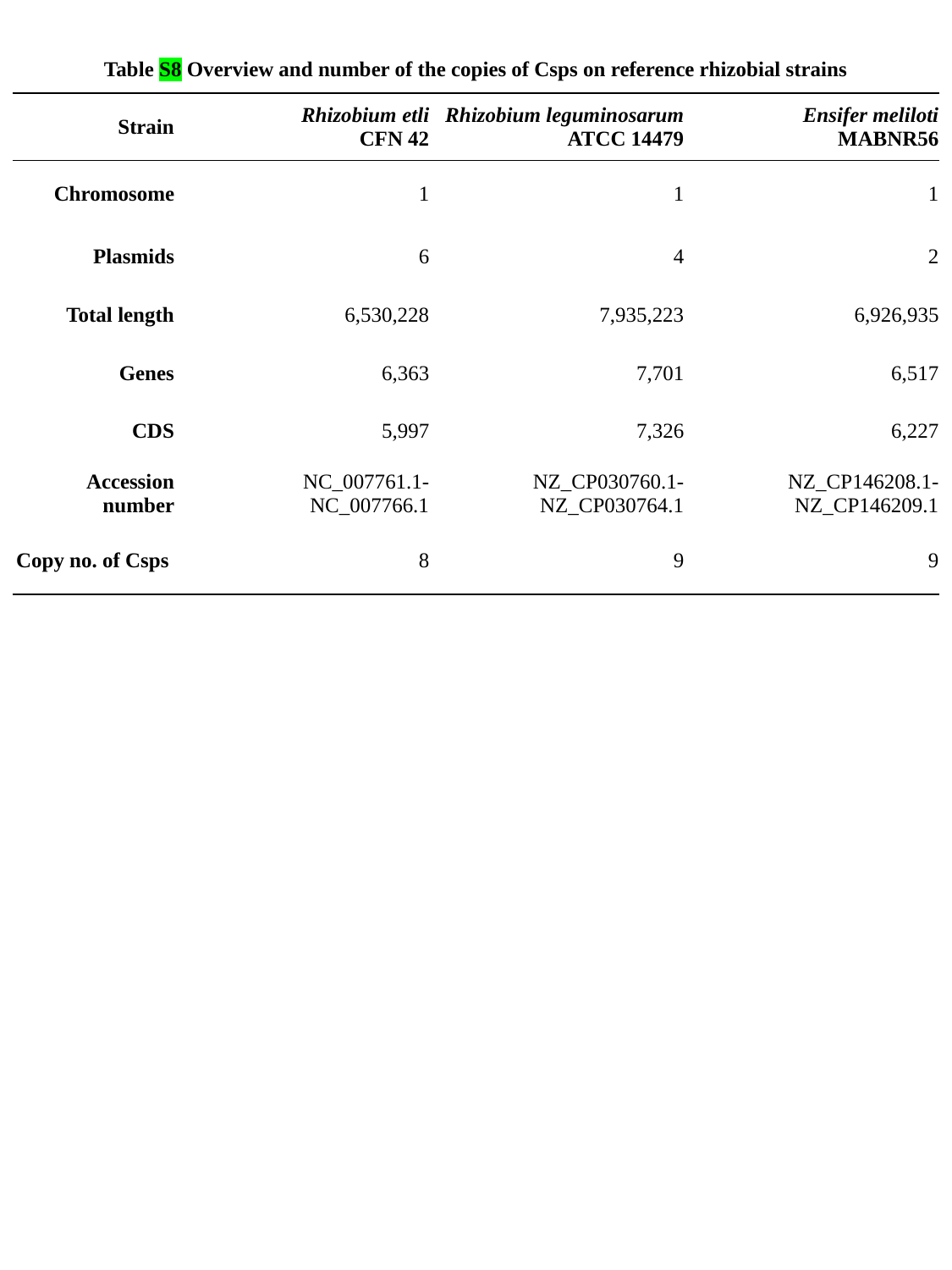

| Table S8 Overview and number of the copies of Csps on reference rhizobial strains | | | |
| --- | --- | --- | --- |
| Strain | Rhizobium etli CFN 42 | Rhizobium leguminosarum ATCC 14479 | Ensifer meliloti  MABNR56 |
| Chromosome | 1 | 1 | 1 |
| Plasmids | 6 | 4 | 2 |
| Total length | 6,530,228 | 7,935,223 | 6,926,935 |
| Genes | 6,363 | 7,701 | 6,517 |
| CDS | 5,997 | 7,326 | 6,227 |
| Accession number | NC\_007761.1- NC\_007766.1 | NZ\_CP030760.1-NZ\_CP030764.1 | NZ\_CP146208.1-NZ\_CP146209.1 |
| Copy no. of Csps | 8 | 9 | 9 |

## Slide 9
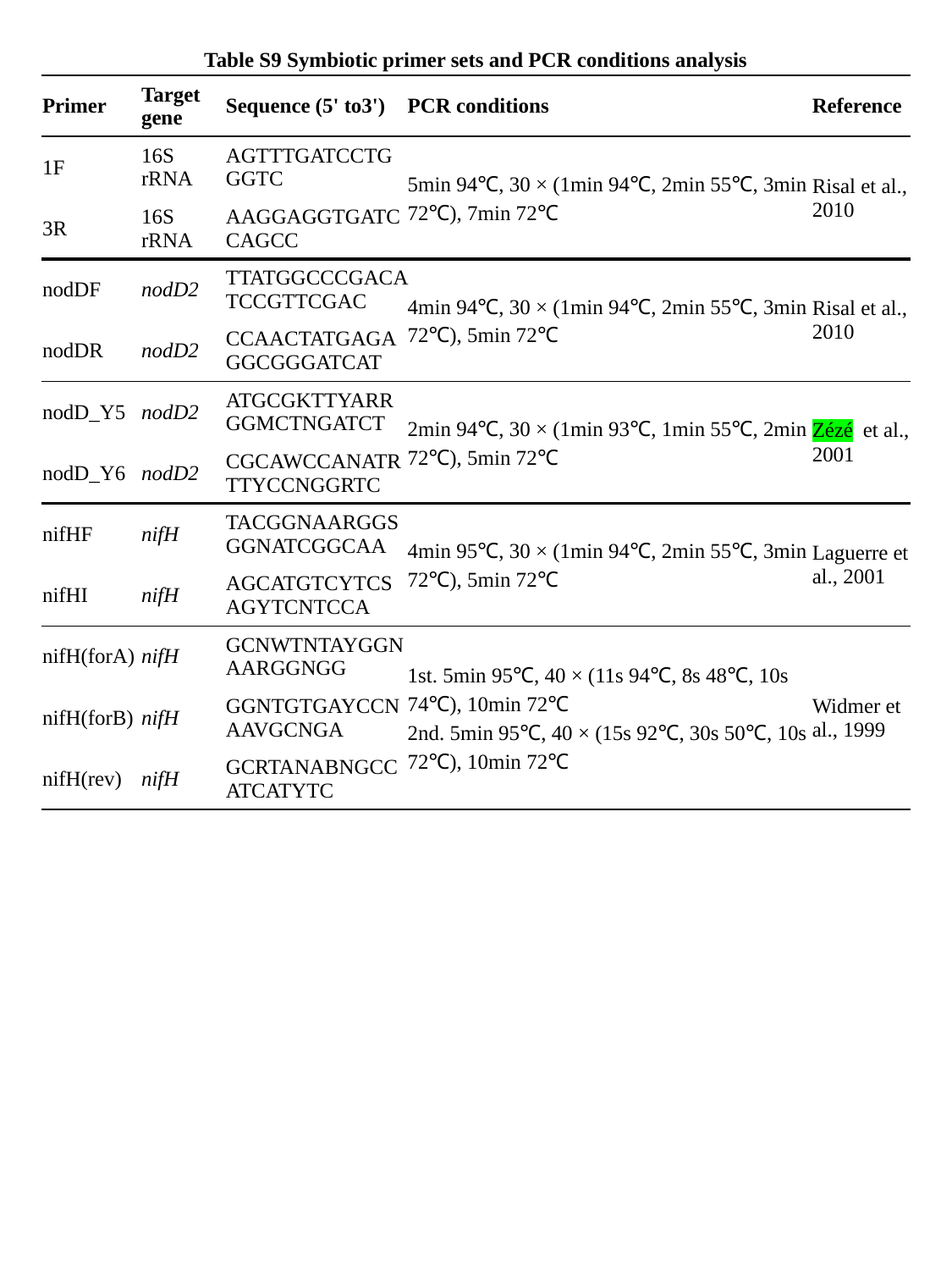

| Table S9 Symbiotic primer sets and PCR conditions analysis | | | | |
| --- | --- | --- | --- | --- |
| Primer | Target gene | Sequence (5' to3') | PCR conditions | Reference |
| 1F | 16S rRNA | AGTTTGATCCTGGGTC | 5min 94℃, 30 × (1min 94℃, 2min 55℃, 3min 72℃), 7min 72℃ | Risal et al., 2010 |
| 3R | 16S rRNA | AAGGAGGTGATCCAGCC | | |
| nodDF | nodD2 | TTATGGCCCGACATCCGTTCGAC | 4min 94℃, 30 × (1min 94℃, 2min 55℃, 3min 72℃), 5min 72℃ | Risal et al., 2010 |
| nodDR | nodD2 | CCAACTATGAGAGGCGGGATCAT | | |
| nodD\_Y5 | nodD2 | ATGCGKTTYARRGGMCTNGATCT | 2min 94℃, 30 × (1min 93℃, 1min 55℃, 2min 72℃), 5min 72℃ | Zézé et al., 2001 |
| nodD\_Y6 | nodD2 | CGCAWCCANATRTTYCCNGGRTC | | |
| nifHF | nifH | TACGGNAARGGSGGNATCGGCAA | 4min 95℃, 30 × (1min 94℃, 2min 55℃, 3min 72℃), 5min 72℃ | Laguerre et al., 2001 |
| nifHI | nifH | AGCATGTCYTCSAGYTCNTCCA | | |
| nifH(forA) | nifH | GCNWTNTAYGGNAARGGNGG | 1st. 5min 95℃, 40 × (11s 94℃, 8s 48℃, 10s 74℃), 10min 72℃ 2nd. 5min 95℃, 40 × (15s 92℃, 30s 50℃, 10s 72℃), 10min 72℃ | Widmer et al., 1999 |
| nifH(forB) | nifH | GGNTGTGAYCCNAAVGCNGA | | |
| nifH(rev) | nifH | GCRTANABNGCCATCATYTC | | |

## Slide 10
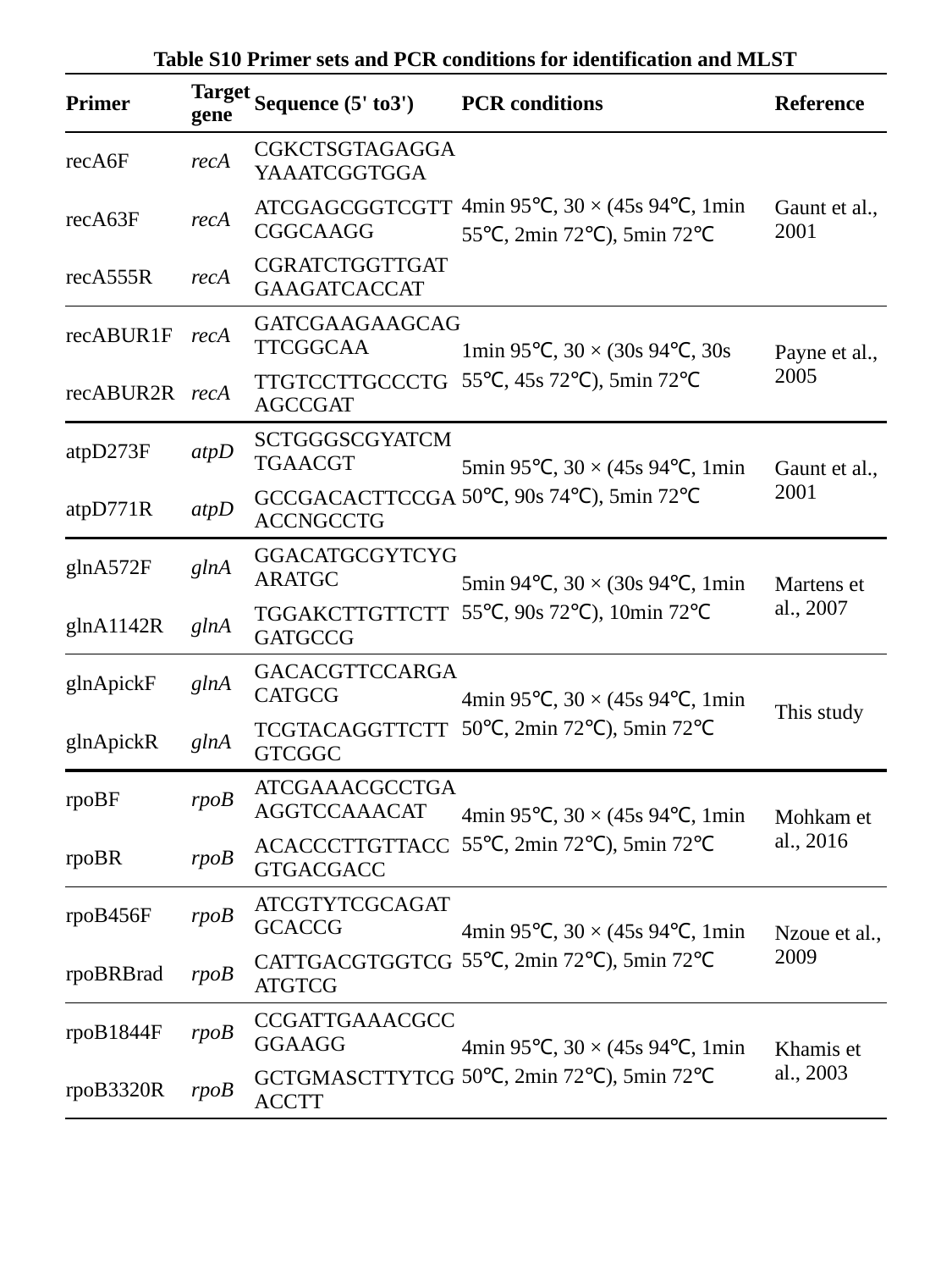

| Table S10 Primer sets and PCR conditions for identification and MLST | | | | |
| --- | --- | --- | --- | --- |
| Primer | Target gene | Sequence (5' to3') | PCR conditions | Reference |
| recA6F | recA | CGKCTSGTAGAGGAYAAATCGGTGGA | 4min 95℃, 30 × (45s 94℃, 1min 55℃, 2min 72℃), 5min 72℃ | Gaunt et al., 2001 |
| recA63F | recA | ATCGAGCGGTCGTTCGGCAAGG | | |
| recA555R | recA | CGRATCTGGTTGATGAAGATCACCAT | | |
| recABUR1F | recA | GATCGAAGAAGCAGTTCGGCAA | 1min 95℃, 30 × (30s 94℃, 30s 55℃, 45s 72℃), 5min 72℃ | Payne et al., 2005 |
| recABUR2R | recA | TTGTCCTTGCCCTGAGCCGAT | | |
| atpD273F | atpD | SCTGGGSCGYATCMTGAACGT | 5min 95℃, 30 × (45s 94℃, 1min 50℃, 90s 74℃), 5min 72℃ | Gaunt et al., 2001 |
| atpD771R | atpD | GCCGACACTTCCGAACCNGCCTG | | |
| glnA572F | glnA | GGACATGCGYTCYGARATGC | 5min 94℃, 30 × (30s 94℃, 1min 55℃, 90s 72℃), 10min 72℃ | Martens et al., 2007 |
| glnA1142R | glnA | TGGAKCTTGTTCTTGATGCCG | | |
| glnApickF | glnA | GACACGTTCCARGACATGCG | 4min 95℃, 30 × (45s 94℃, 1min 50℃, 2min 72℃), 5min 72℃ | This study |
| glnApickR | glnA | TCGTACAGGTTCTTGTCGGC | | |
| rpoBF | rpoB | ATCGAAACGCCTGAAGGTCCAAACAT | 4min 95℃, 30 × (45s 94℃, 1min 55℃, 2min 72℃), 5min 72℃ | Mohkam et al., 2016 |
| rpoBR | rpoB | ACACCCTTGTTACCGTGACGACC | | |
| rpoB456F | rpoB | ATCGTYTCGCAGATGCACCG | 4min 95℃, 30 × (45s 94℃, 1min 55℃, 2min 72℃), 5min 72℃ | Nzoue et al., 2009 |
| rpoBRBrad | rpoB | CATTGACGTGGTCGATGTCG | | |
| rpoB1844F | rpoB | CCGATTGAAACGCCGGAAGG | 4min 95℃, 30 × (45s 94℃, 1min 50℃, 2min 72℃), 5min 72℃ | Khamis et al., 2003 |
| rpoB3320R | rpoB | GCTGMASCTTYTCGACCTT | | |

## Slide 11
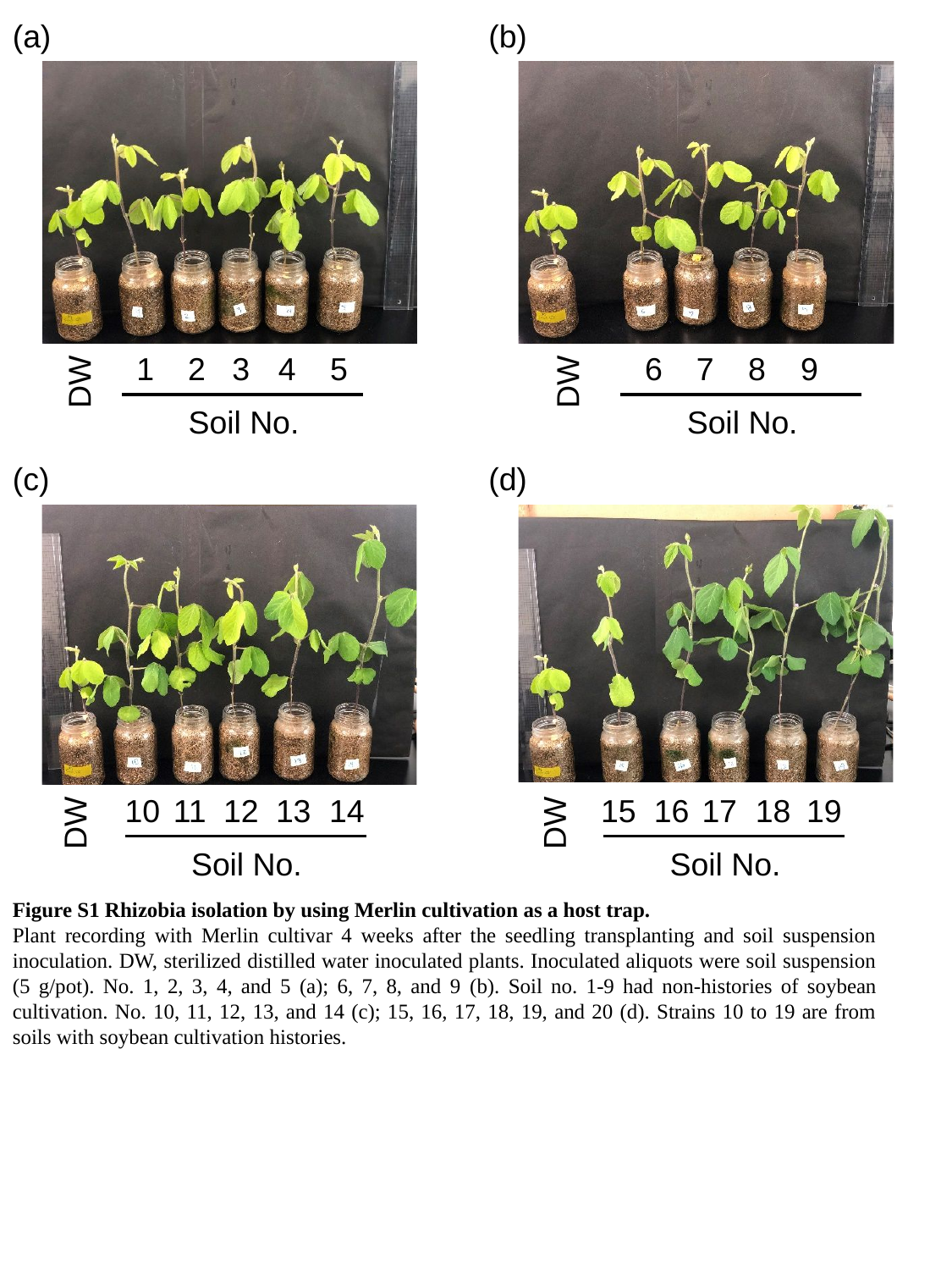

(a)
(b)
1
2
3
4
5
6
7
8
9
DW
DW
Soil No.
Soil No.
(c)
(d)
10
11
12
13
14
15
16
17
18
19
DW
DW
Soil No.
Soil No.
Figure S1 Rhizobia isolation by using Merlin cultivation as a host trap.
Plant recording with Merlin cultivar 4 weeks after the seedling transplanting and soil suspension inoculation. DW, sterilized distilled water inoculated plants. Inoculated aliquots were soil suspension (5 g/pot). No. 1, 2, 3, 4, and 5 (a); 6, 7, 8, and 9 (b). Soil no. 1-9 had non-histories of soybean cultivation. No. 10, 11, 12, 13, and 14 (c); 15, 16, 17, 18, 19, and 20 (d). Strains 10 to 19 are from soils with soybean cultivation histories.

## Slide 12
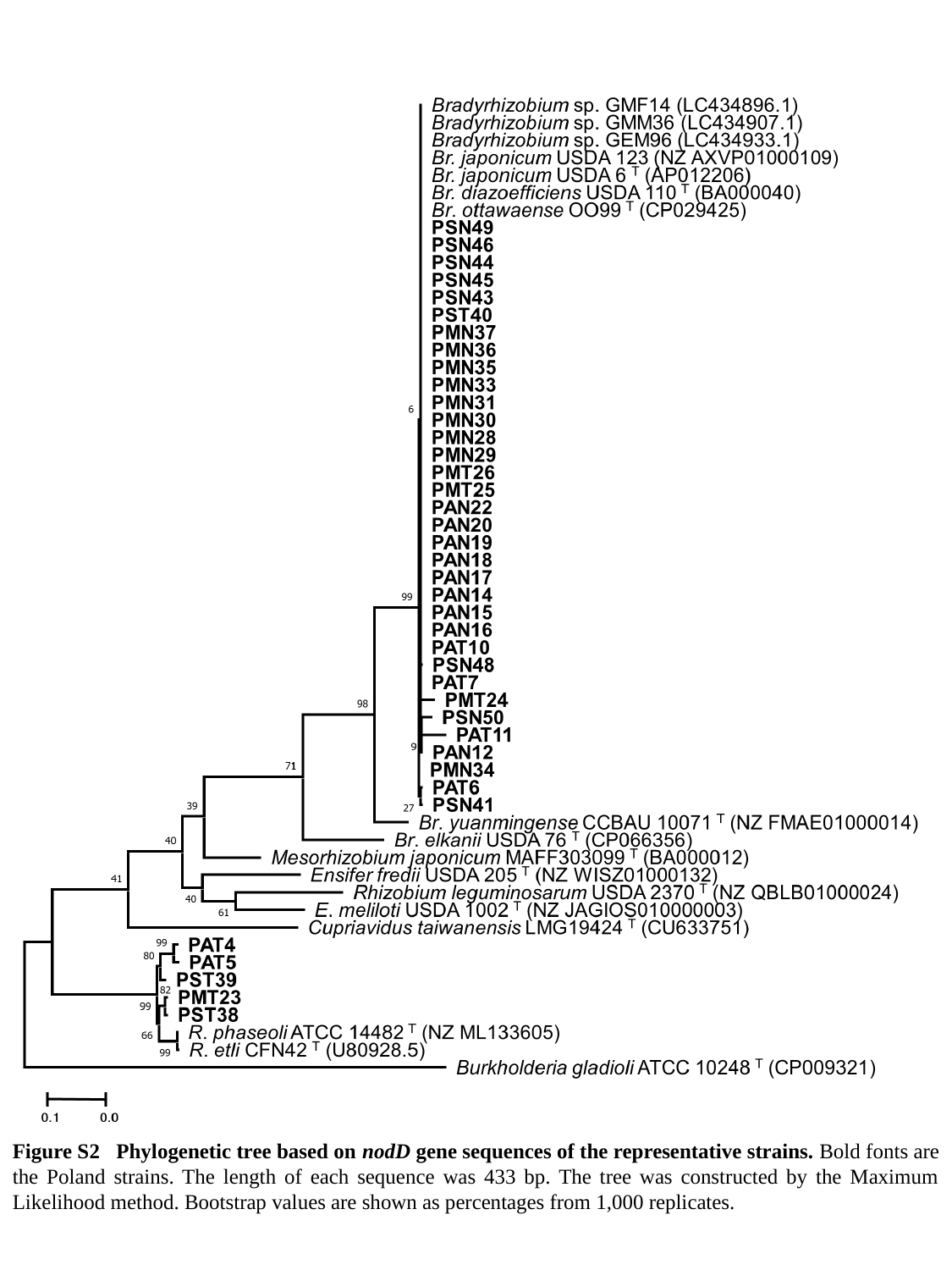

Figure S2 Phylogenetic tree based on nodD gene sequences of the representative strains. Bold fonts are the Poland strains. The length of each sequence was 433 bp. The tree was constructed by the Maximum Likelihood method. Bootstrap values are shown as percentages from 1,000 replicates.

## Slide 13
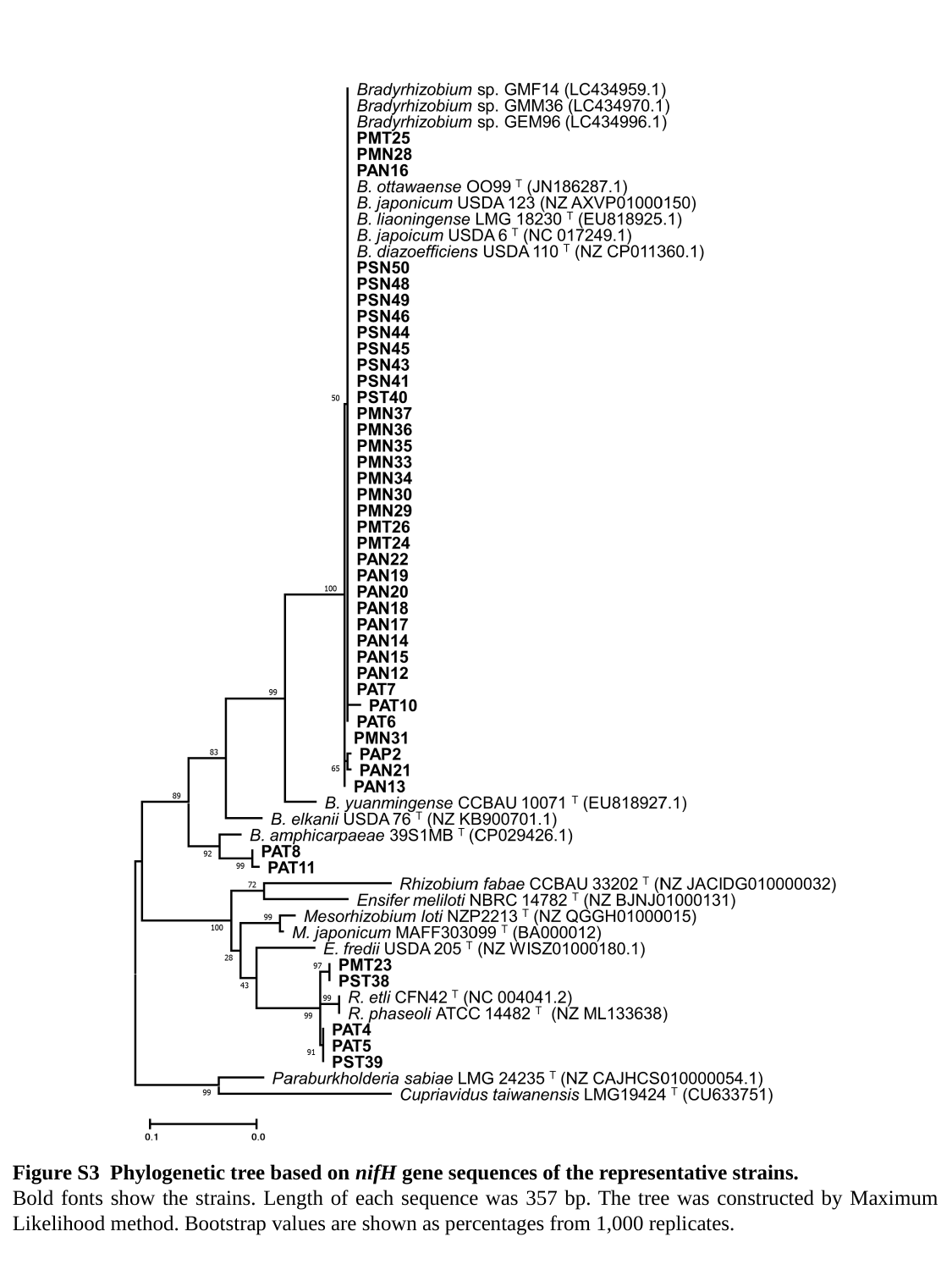

Figure S3 Phylogenetic tree based on nifH gene sequences of the representative strains.
Bold fonts show the strains. Length of each sequence was 357 bp. The tree was constructed by Maximum Likelihood method. Bootstrap values are shown as percentages from 1,000 replicates.

## Slide 14
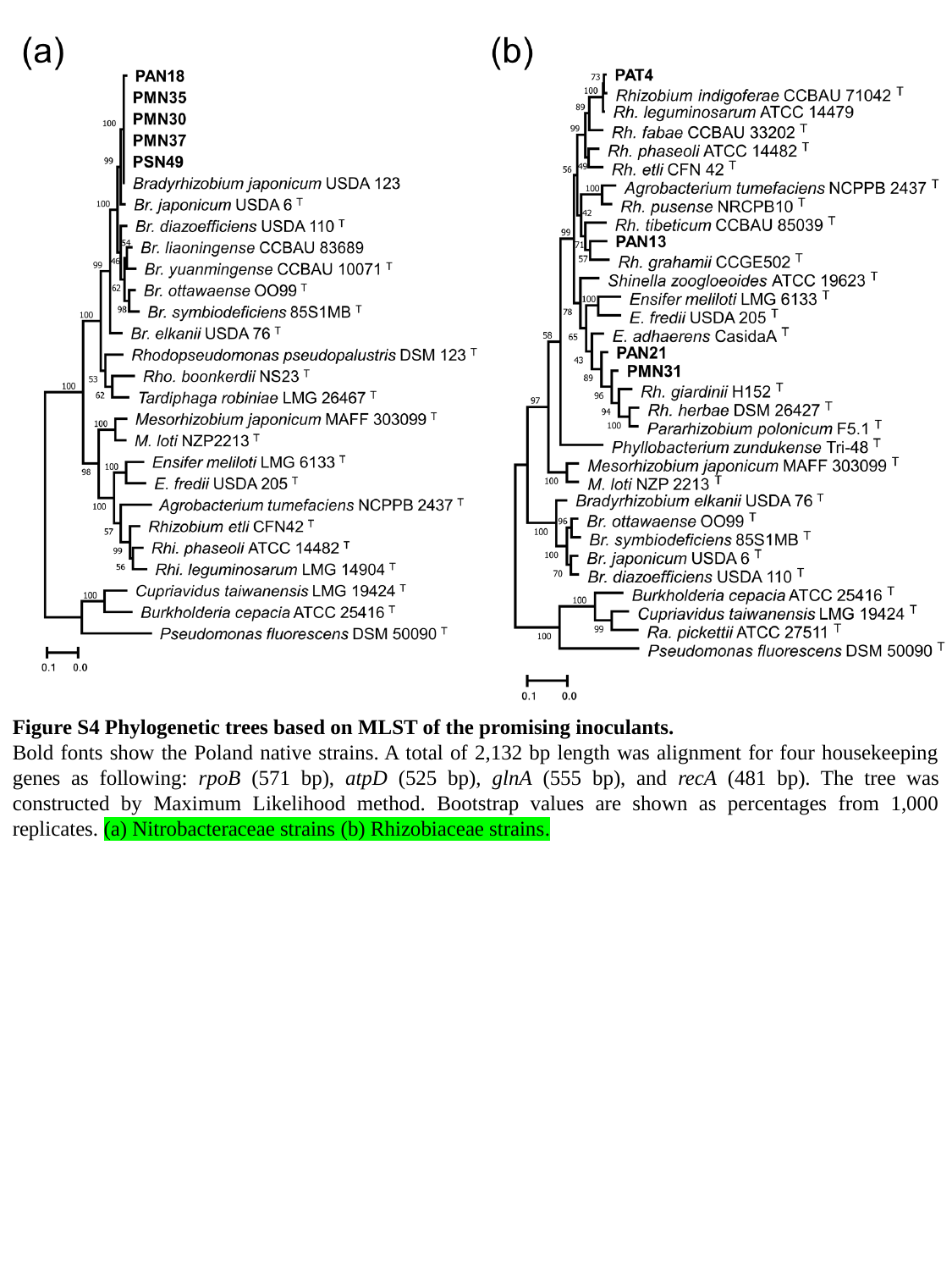

Figure S4 Phylogenetic trees based on MLST of the promising inoculants.
Bold fonts show the Poland native strains. A total of 2,132 bp length was alignment for four housekeeping genes as following: rpoB (571 bp), atpD (525 bp), glnA (555 bp), and recA (481 bp). The tree was constructed by Maximum Likelihood method. Bootstrap values are shown as percentages from 1,000 replicates. (a) Nitrobacteraceae strains (b) Rhizobiaceae strains.

## Slide 15
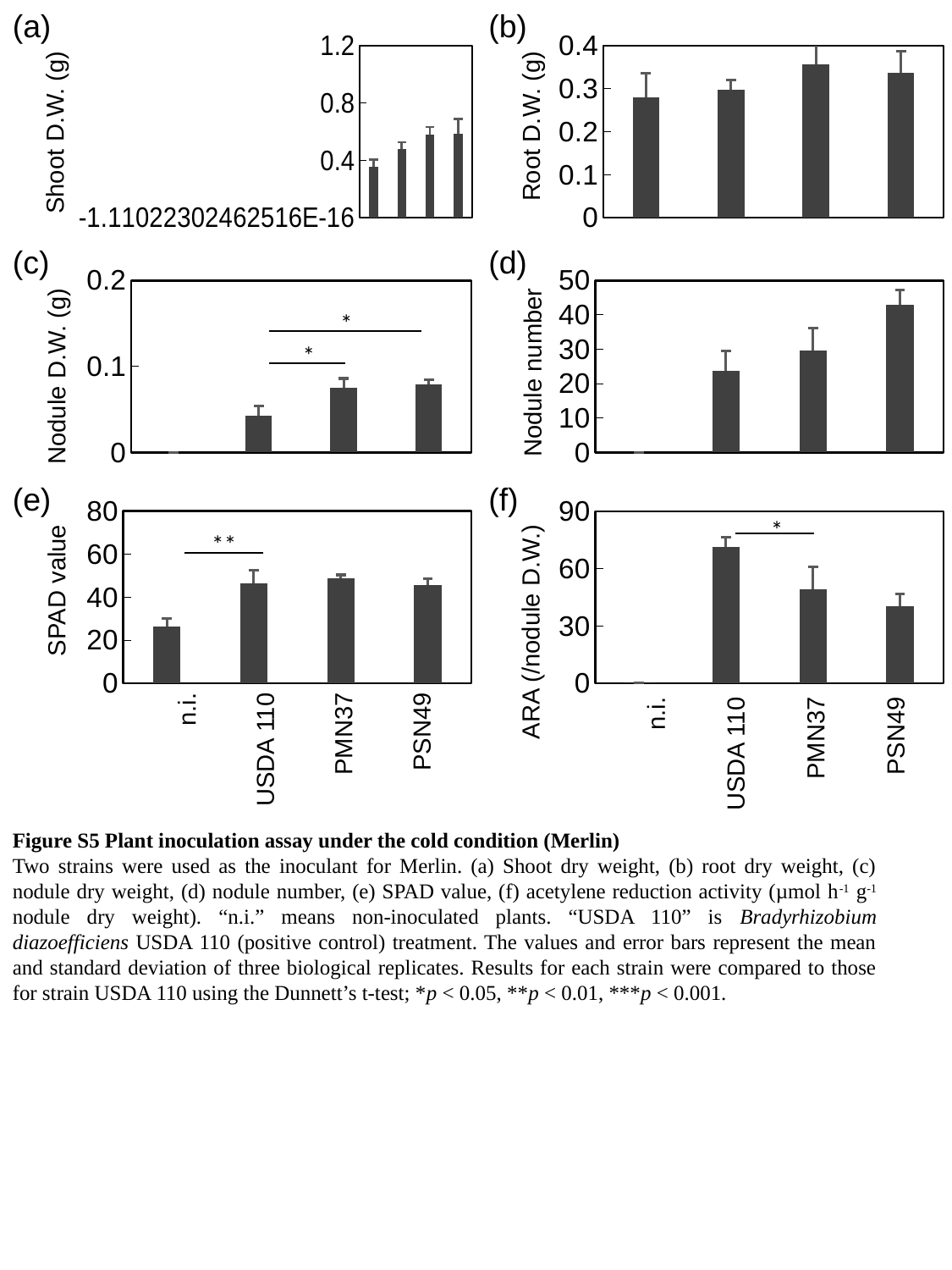

(a)
(b)
### Chart
| Category | |
|---|---|
| n.i. | 0.3568 |
| B. USDA110 | 0.4796 |
| B. MN37 | 0.5804 |
| B. SN49 | 0.5839 |
### Chart
| Category | |
|---|---|
| n.i. | 0.2801 |
| B. USDA110 | 0.2970333333333333 |
| B. MN37 | 0.3562 |
| B. SN49 | 0.3360666666666667 |Shoot D.W. (g)
Root D.W. (g)
(c)
(d)
### Chart
| Category | |
|---|---|
| n.i. | 0.0 |
| B. USDA110 | 0.042633333333333336 |
| B. MN37 | 0.07506666666666666 |
| B. SN49 | 0.0792 |
### Chart
| Category | |
|---|---|
| n.i. | 0.0 |
| B. USDA110 | 23.666666666666668 |
| B. MN37 | 29.666666666666668 |
| B. SN49 | 43.0 |*
*
Nodule D.W. (g)
Nodule number
(e)
(f)
### Chart
| Category | |
|---|---|
| n.i. | 26.26666666666667 |
| B. USDA110 | 46.43333333333334 |
| B. MN37 | 48.93333333333334 |
| B. SN49 | 45.76666666666667 |
### Chart
| Category | |
|---|---|
| n.i. | 0.0 |
| B. USDA110 | 71.39538713808908 |
| B. MN37 | 49.23893223071071 |
| B. SN49 | 40.346048078700264 |*
**
SPAD value
ARA (/nodule D.W.)
n.i.
USDA 110
PMN37
PSN49
n.i.
USDA 110
PMN37
PSN49
Figure S5 Plant inoculation assay under the cold condition (Merlin)
Two strains were used as the inoculant for Merlin. (a) Shoot dry weight, (b) root dry weight, (c) nodule dry weight, (d) nodule number, (e) SPAD value, (f) acetylene reduction activity (µmol h-1 g-1 nodule dry weight). “n.i.” means non-inoculated plants. “USDA 110” is Bradyrhizobium diazoefficiens USDA 110 (positive control) treatment. The values and error bars represent the mean and standard deviation of three biological replicates. Results for each strain were compared to those for strain USDA 110 using the Dunnett’s t-test; *p < 0.05, **p < 0.01, ***p < 0.001.

## Slide 16
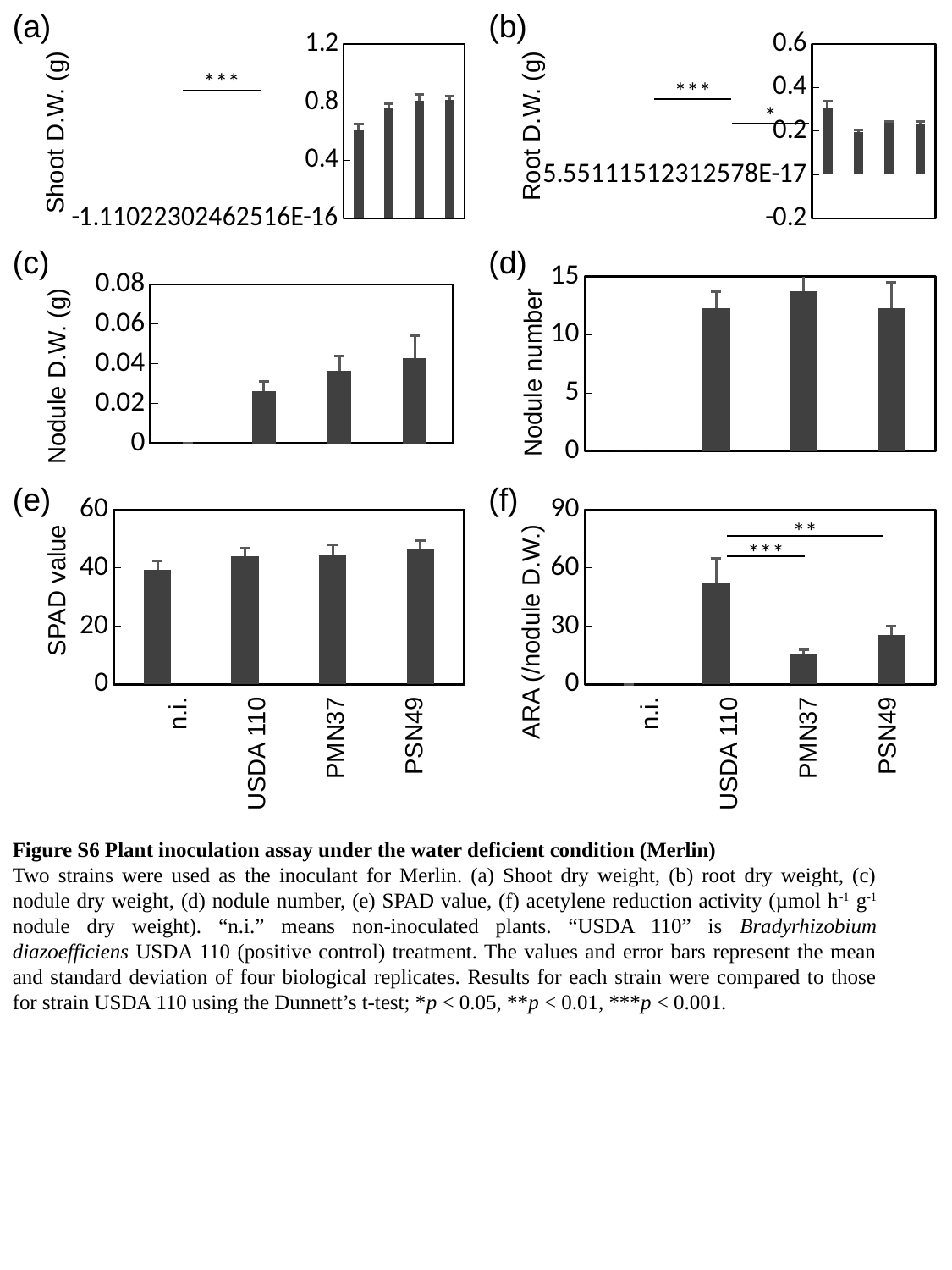

(a)
(b)
### Chart
| Category | |
|---|---|
| n.i. | 0.6025750000000001 |
| B. USDA110 | 0.7606499999999999 |
| B. MN37 | 0.8112 |
| B. SN49 | 0.815625 |
### Chart
| Category | |
|---|---|
| n.i. | 0.307575 |
| B. USDA110 | 0.19492500000000001 |
| B. MN37 | 0.237025 |
| B. SN49 | 0.23107500000000003 |***
***
*
Shoot D.W. (g)
Root D.W. (g)
(c)
(d)
### Chart
| Category | |
|---|---|
| n.i. | 0.0 |
| B. USDA110 | 0.026175 |
| B. MN37 | 0.036325 |
| B. SN49 | 0.043050000000000005 |
### Chart
| Category | |
|---|---|
| n.i. | 0.0 |
| B. USDA110 | 12.25 |
| B. MN37 | 13.75 |
| B. SN49 | 12.25 |Nodule D.W. (g)
Nodule number
(e)
(f)
### Chart
| Category | |
|---|---|
| n.i. | 39.375 |
| B. USDA110 | 44.099999999999994 |
| B. MN37 | 44.7 |
| B. SN49 | 46.375 |
### Chart
| Category | |
|---|---|
| ac- | 0.0 |
| ac+ | 52.38474206656195 |
| a4 | 15.82648992160357 |
| a5 | 25.6171526535445 |**
***
SPAD value
ARA (/nodule D.W.)
n.i.
USDA 110
PMN37
PSN49
n.i.
USDA 110
PMN37
PSN49
Figure S6 Plant inoculation assay under the water deficient condition (Merlin)
Two strains were used as the inoculant for Merlin. (a) Shoot dry weight, (b) root dry weight, (c) nodule dry weight, (d) nodule number, (e) SPAD value, (f) acetylene reduction activity (µmol h-1 g-1 nodule dry weight). “n.i.” means non-inoculated plants. “USDA 110” is Bradyrhizobium diazoefficiens USDA 110 (positive control) treatment. The values and error bars represent the mean and standard deviation of four biological replicates. Results for each strain were compared to those for strain USDA 110 using the Dunnett’s t-test; *p < 0.05, **p < 0.01, ***p < 0.001.
